# Tailored Bisacylphosphane Oxides for Precise Induction of Oxidative Stress-Mediated Cell Death in Biological Systems

**DOI:** 10.1021/acschembio.4c00399

**Published:** 2024-11-05

**Authors:** Karim Almahayni, Jana Bachir Salvador, Riccardo Conti, Anna Widera, Malte Spiekermann, Daniel Wehner, Hansjörg Grützmacher, Leonhard Möckl

**Affiliations:** 1 Max Planck Institute for the Science of Light, Staudtstr. 2, 91058 Erlangen, Germany; 2 Department of Physics, Friedrich-Alexander-University Erlangen-Nürnberg, Staudtstr. 5, 91054 Erlangen, Germany; 3 Max-Planck-Zentrum für Physik und Medizin, Staudtstr. 2, 91054 Erlangen, Germany; 4 Department of Chemistry and Applied Biosciences, ETH Zurich, Vladimir-Prelog-Weg 1, 8093 Zurich, Switzerland; 5 Department of Chemistry and Pharmacy, Inorganic Chemistry, Friedrich-Alexander-University Erlangen-Nürnberg, Egerlandstr. 1, 91058 Erlangen, Germany; 6 Department of Medicine 1/CITABLE, Friedrich-Alexander-University Erlangen-Nürnberg, Ulmenweg 18, D-91054 Erlangen, Germany; 7 Deutsches Zentrum Immuntherapie (DZI), Uniklinikum Erlangen, 91054 Erlangen,Germany

## Abstract

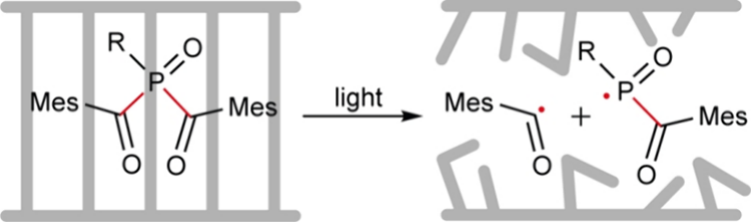

Precise cell elimination within intricate cellular populations is hampered by issues arising from the multifaceted biological properties of cells and the expansive reactivity of chemical agents. Current chemical platforms are often limited by their complexity, toxicity, and poor physical/chemical properties. Here, we report on the synthesis of a structurally versatile library of chemically tunable bisacylphosphane oxides (BAPOs), which harnesses the spatiotemporal precision of light delivery, thereby establishing a universal strategy for on-demand, precise cellular ablation *in vitro* and *in vivo*.

## Introduction

Precise elimination of selected cells from a dense cell population remains a challenging task. This is attributable to the heterogeneous nature of cells and, in contrast to that, the broad and systemic activity of chemical compounds such as chemotherapeutics. Strategies to ablate target cells have found various applications, for example in studies of cancer, aging, and tissue regeneration.^1−3^ Especially light has been used in combination with chemical or genetic probes as a tool to enable targeted cell ablation (photochemical and optogenetic strategies, respectively), among other applications like neuronal activation.^4−7^ While genetic incorporation of photoactivatable proteins can be advantageous in terms of specificity, the generation of transgenic model systems is often costly, time-intensive, and generally challenging to implement in a clinical setting. Photochemical agents exogenously supplied to cell populations or whole organisms can provide a faster and cheaper alternative. However, the biological use of conventional photosensitizers is often hampered by issues related to water solubility, oxygen dependency, degradation, off-target distribution, and undesired immunogenicity.^8^

Phototoxic compounds with properties tailored for applications in chemical biology and biochemistry should (i) easily dissolve in aqueous media, (ii) exhibit minimal toxicity in the dark, (iii) be able to be activated at low irradiation doses without provoking light toxicity, (iv) exhibit an oxygen-independent mode of action, and (v) convert into a physiological inactive form after photolysis to avoid unwanted long-term effects. In this work, we introduce a class of compounds that meets these criteria. We characterize their working mechanism and establish their application in biological systems.

**Figure 1:**
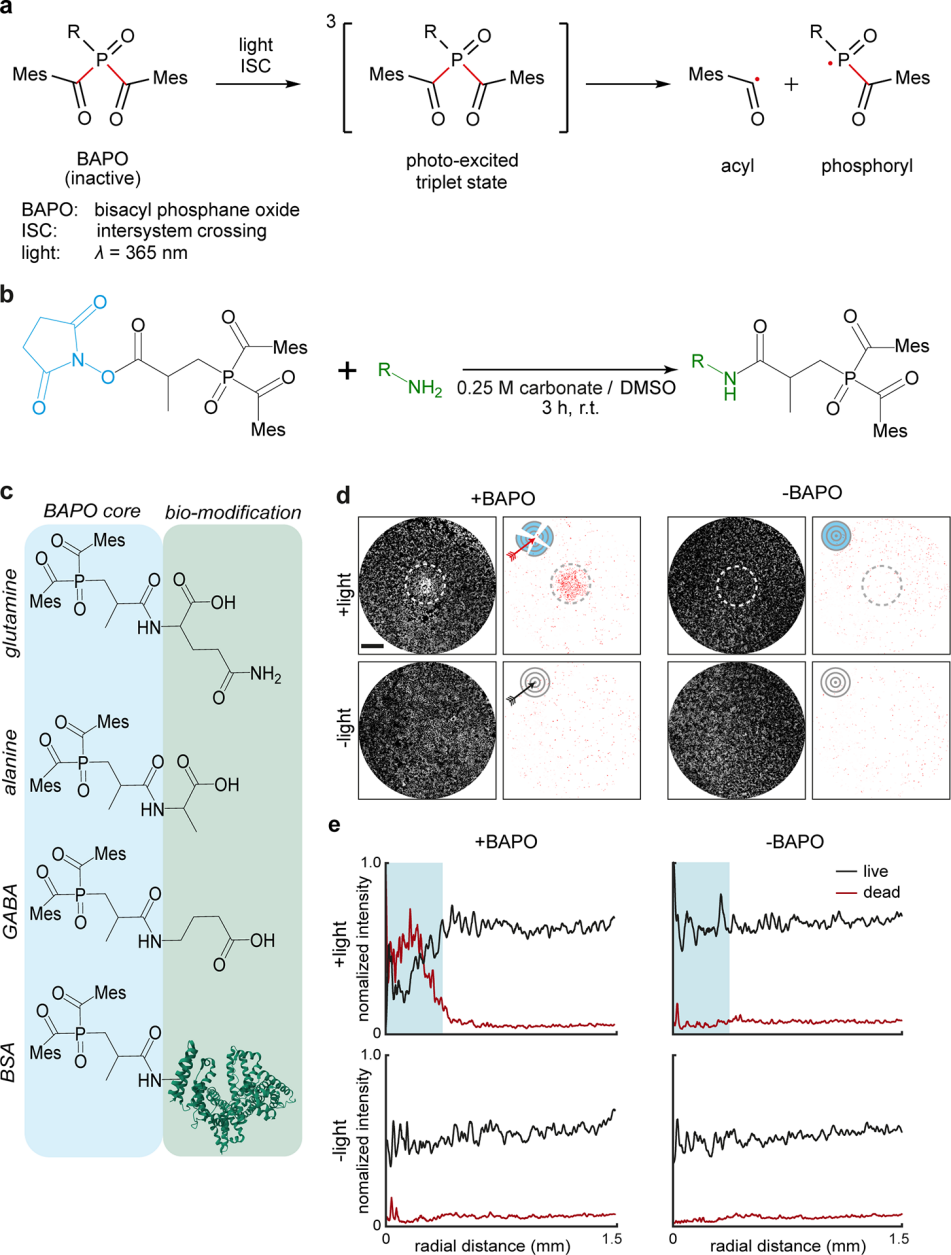
Concept, chemistry, and characterization of BIOS. (a) Mechanism of BAPO activation. One bond cleavage is shown. Another pair of radicals can be generated by cleavage of the second bond highlighted in red. Mes = mesitoyl group. (b) Coupling of NHS-BAPO to a target amine. The NHS group is shown in cyan, and the target amine in green. (c) Library of BAPO conjugates synthesized and investigated. Cyan shading denotes the BAPO core and green shading the biomodification. BSA is shown as a cartoon representation of the protein structure (PDB ID: 3V03). (d) BIOS induces local, controlled cell death upon light activation (upper left) and exhibits no dark toxicity (lower left) or light toxicity (upper right) compared to the untreated and unirradiated control (lower right). Live cells of each condition are displayed on the left panel (inverted LUT, black is high signal), and the dead cells are displayed on the right panel (red). In the cartoons, BAPOs are represented by an arrow (red = activation, gray = no activation), the cells are shown as a target, and light is depicted in blue. The irradiation area for the light only condition is highlighted via dashed circles. Scale bar: 500 μm. Intensity: 9 mW/cm^2^. Calcein A/ethidium homodimers were used to identify live/dead cells, respectively. BAPO used: alanine-BAPO, 60 μM. Cell line: A549. (e) Radial profiles around the center of the well, corresponding to the images shown in panel (d). For the two irradiated conditions, the irradiation area is depicted via light blue shading. Black line: signal from live cells; red line: signal from dead cells. All curves are normalized to the maximum signal across the different conditions for the live and dead signal, respectively. For the BAPO-treated and light-irradiated conditions, the live signal decreases and the dead signal increases in the activation area. No effect is observed for the other conditions.

Bisacylphosphane oxides (BAPOs) form a family of photoactivatable molecules with documented applications in industry and dentistry. Examples include lacquer curing and dental fillings^9,10^ Chemically, BAPOs belong to the class of Norrish type I photolatent compounds.^11^ After light irradiation with approximately 365 nm light at biocompatible dosage, they directly decay into radicals in a first-order reaction without the need for a co-initiator. This process is initiated by excitation into the first singlet state. Then, intersystem crossing occurs as the rate-determining step, yielding a short-lived triplet state.^12^ After this, rapid homolytic cleavage of the acyl-phosphorus bond creates an acyl and a phosphoryl radical.^13^ As BAPOs contain two such bonds, consecutive cleavage may generate up to four radical species (Figure 1a).

Building on the photochemical potential of BAPOs, we report the synthesis of a library of functionalized BAPO compounds that are optimized for safety, cell permeability, stability, and efficacy. We demonstrate that this customizable chemical platform in combination with the spatiotemporal precision of light delivery allows for robust ablation of target cells *in vitro* and *in vivo* on demand. We coin this strategy as BIOS (BAPO-mediated Induction of Oxidative Stress).

## Results and Discussion

### Concept, Platform, and Chemical Synthesis

Our decision to employ BAPOs is driven by their favorable features compared to other photolatent species: (i) they can be chemically tuned without affecting the photoactive core and thus be readily tailored to the desired application, (ii) they can be activated with light of comparably low energy in the range of 350–400 nm, which minimizes potential tissue damages while allowing adequate penetration depths and activation of BAPOs in tissue,^14^ (iii) BAPOs are activated once, avoiding unwanted long-term effects,^12^ (iv) the radical generation mechanism of BAPOs is oxygen-independent, which provides an advantage over oxygen-dependent photosensitizers,^15^ and (v) studies from our group and others revealed that BAPOs show negligible cytotoxicity and can be applied for photoactivation in aqueous media where conventional photolatent compounds are insoluble.^16^

To make use of these properties in order to establish a platform of BAPO molecules, we synthesized a reactive BAPO analogue, *N*-hydroxy succinimide-BAPO (NHS-BAPO). The synthesis of this BAPO derivative is performed using *N*-succinimidyl methacrylate as a Michael acceptor and BAP-H as a Michael donor.^17^ The subsequent ligation of NHS-BAPO is straightforward. The target substrate bearing a primary amine (0.75 mmol, 1.3 equiv) is dissolved in 6 mL of 0.25 M sodium carbonate buffer (corresponding to 1.43 mmol, 2.5 equiv of base) in a 25 mL round-bottomed flask. NHS-BAPO (300 mg, 0.57 mmol, 1 equiv) is dissolved in 2 mL of DMSO in a vial protected from light. The NHS-BAPO solution of NHS-BAPO is added dropwise to the aqueous solution under vigorous stirring. Stirring is continued for 3 h at RT, protected from light. The mixture is then filtered and transferred to a separating funnel; 15 mL of dichloromethane (DCM) is added followed by 15 mL of 2 M HCl. After the separation, the organic phase is washed 2 times with 15 mL of brine. The collected brine phases are washed with 15 mL of DCM. The collected organic phases are dried using Na_2_CO_3_. After the drying agent is filtered, the solvent is evaporated and the product isolated (Figure 1b).

Primary amines are highly nucleophilic and therefore readily react with NHS esters, forming stable conjugates.^18^ As primary amines are present in almost all proteins and many relevant biomolecules, it is straightforward to link them to BAPOs via this approach. We highlight the low synthetic complexity of this platform, allowing for the straightforward dissemination of the approach in other laboratories.

Exploiting the versatility of NHS-BAPO, we synthesized a library of functionalized BAPOs with improved water solubility, cellular uptake, and targeting capabilities. We screened an array of common biological molecules, taking inspiration from previous drug delivery studies.^19−21^ Exploring both active and passive targeting, four compounds stood out in our screens: BAPOs modified with glutamine, β-alanine, γ-aminobutyric acid, and bovine serum albumin (BSA) (Figure 1c, Figures S1–S4, and Supporting Information on spectroscopy). Such chemical modifications can prolong circulation and elevate drug concentration at desired sites, thus minimizing toxicity and enhancing efficacy.^22^ The list of compounds created by us is not exhaustive and is straightforward to expand. Furthermore, purification only requires commercially available size exclusion chromatography kits, which are commonly used for, e.g., the isolation of dye-coupled antibodies.

BIOS allows for precise ablation of target cells within a population of cells in a controlled, safe, and efficacious fashion (Figure 1d,e and Figure S5). In Figure 1d, the entire well was treated with alanine-BAPO; however, BIOS is only triggered in the irradiated center of the well (quantified in Figure 1e). Live and dead cells were detected with calcein AM (CalA) and ethidium homodimer-1 (EHD), respectively. Dying cells rapidly lose CalA signal and develop EHD signal on the time scale of few minutes, as recently demonstrated.^23^ For the other conditions, low EHD signal and constantly high Cal-A signal are observed throughout the whole well, indicating no induction of toxicity by the BAPO or the light irradiation alone.

With respect to triggering BIOS, a range of light delivery methods can be employed. To demonstrate this, this study uses irradiation via a custom irradiation device, via directing the light through the objective of a commercial confocal microscope, and via an optical fiber-based design (see Methods and Figures S6 and S7). Notably, alternative light delivery methods can also be implemented, based on the respective use case. To determine an effective light dose by which BIOS can be safely deployed, we screened the efficacy and toxicity profiles at six different time points and two intensities (Figure S8). While a light dose of 53 mW/cm^2^ induced light toxicity after 10 min, a dose of 17 mW/cm^2^ was safe and efficacious over a 45 min period. Thus, we opted for a lower dosage of 15 mW/cm^2^ for 30 min, which was used to activate the BIOS in the characterization screens. For local light delivery via a microscope objective, we employed a dosage of 9 mW/cm^2^ for 30 min.

In this context, it is important to consider the optical characteristics of tissue, which may have a significant influence on the efficiency of light-based compound activation. This is due to the turbidity of tissue, where numerous optical phenomena occur, in particular scattering and absorption. Understanding how light interacts with and is attenuated by tissue is crucial to optimizing BIOS efficacy and safety. To mimic such an application scenario, we investigated the activation of BIOS in the presence of tissue phantoms. We constructed an in *vitro* system in which lung cancer cells (A549) are covered by tissue phantoms that were designed to have similar optical and elastic properties to lung tissue.^24^ We demonstrated that the attenuated intensity can be compensated by prolonged irradiation, not reducing the BIOS efficacy while retaining the absence of light toxicity (Figure S9).

### BIOS Triggers Apoptosis In Vitro

To investigate the safety and efficacy of the BAPO compound library, we applied BIOS to four cell lines. We opted for highly tumorigenic cell lines that represent prevalent cancer types with reported elevated resistance to phototherapy (lung cancer-derived A549, liver cancer-derived Hep3B, and pancreatic cancer-derived PANC-1).^25,26^ In addition, we included noncancerous HMECs (a human microvascular epithelial cell line). This selection reduces the risk of observing cell line-specific effects.

Cells were incubated with different concentrations of the respective compound for 24 h, during which cellular uptake occurs. The cells were then thoroughly washed to ensure that any observed effect was solely caused by internalized compound rather than excess in the media. The cells were then irradiated with 15 mW/cm^2^ (365 nm) for 30 min with the system described in Figure S6. Following irradiation, the cells were returned to the incubator and further cultured for 24 h. Putative light toxicity (such as DNA damage due to light irradiation, for example) or dark toxicity by the inactive compound alone would manifest after this time window. Afterward, live and dead cells were detected with CalA and EHD as described above, respectively. Thus, we achieve an accurate estimate of cell viability, which is not skewed by a delayed response of the reporter.

**Figure 2:**
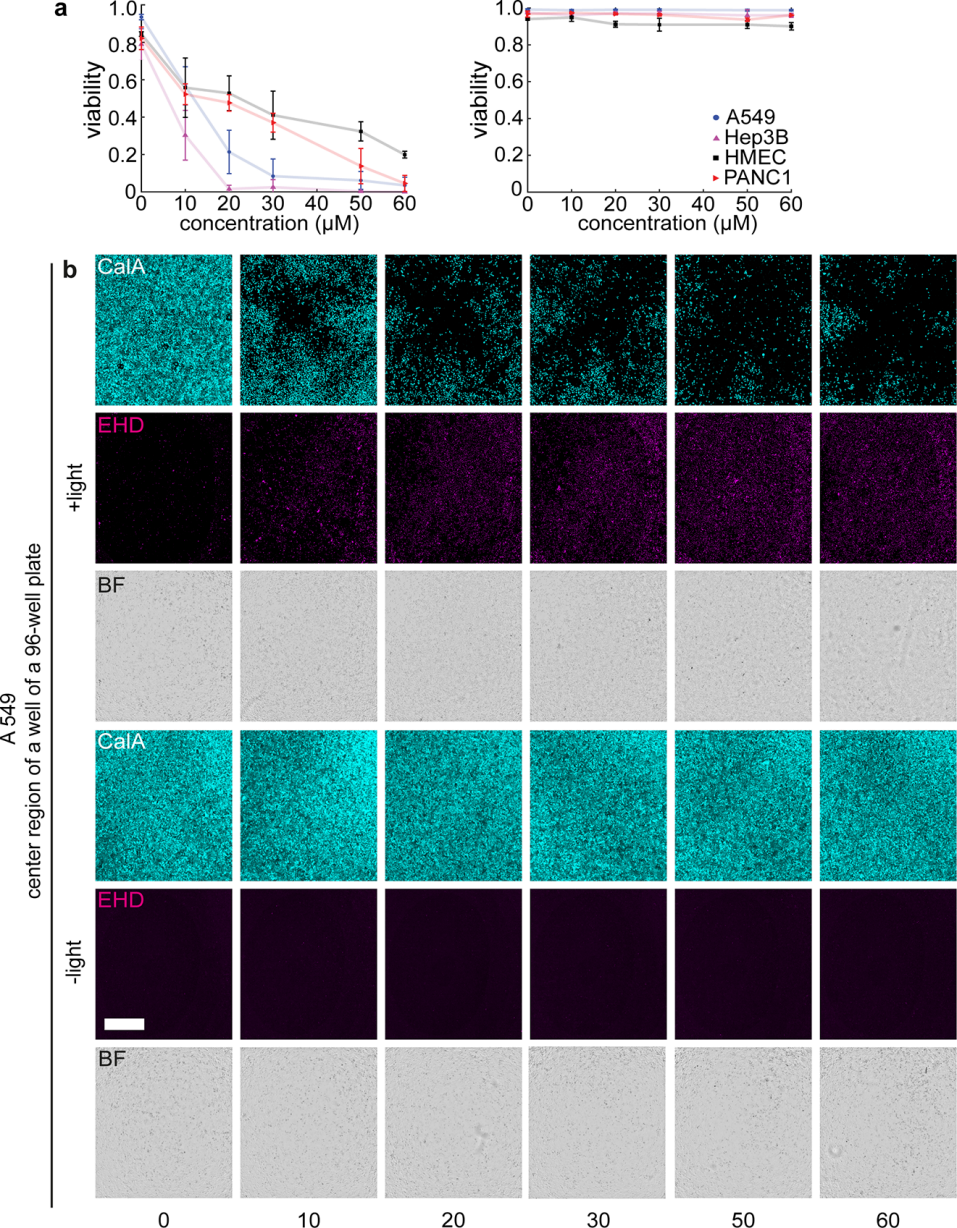
*In vitro* characterization of BIOS. (a) Quantification of cellular viability for the investigated cell lines upon treatment with BSA-BAPO at the indicated concentrations and either subjected to light irradiation or not (left/right panel, respectively). *N* = 3. Error bars: SD. (b) Representative images of A549 cells when treated with BSA-BAPO and either subjected to light activation or not. Upper row: CalA stain (live cells, cyan); bottom row: EHD (dead cells, magenta). Scale bar: 1 mm.

**Figure 3:**
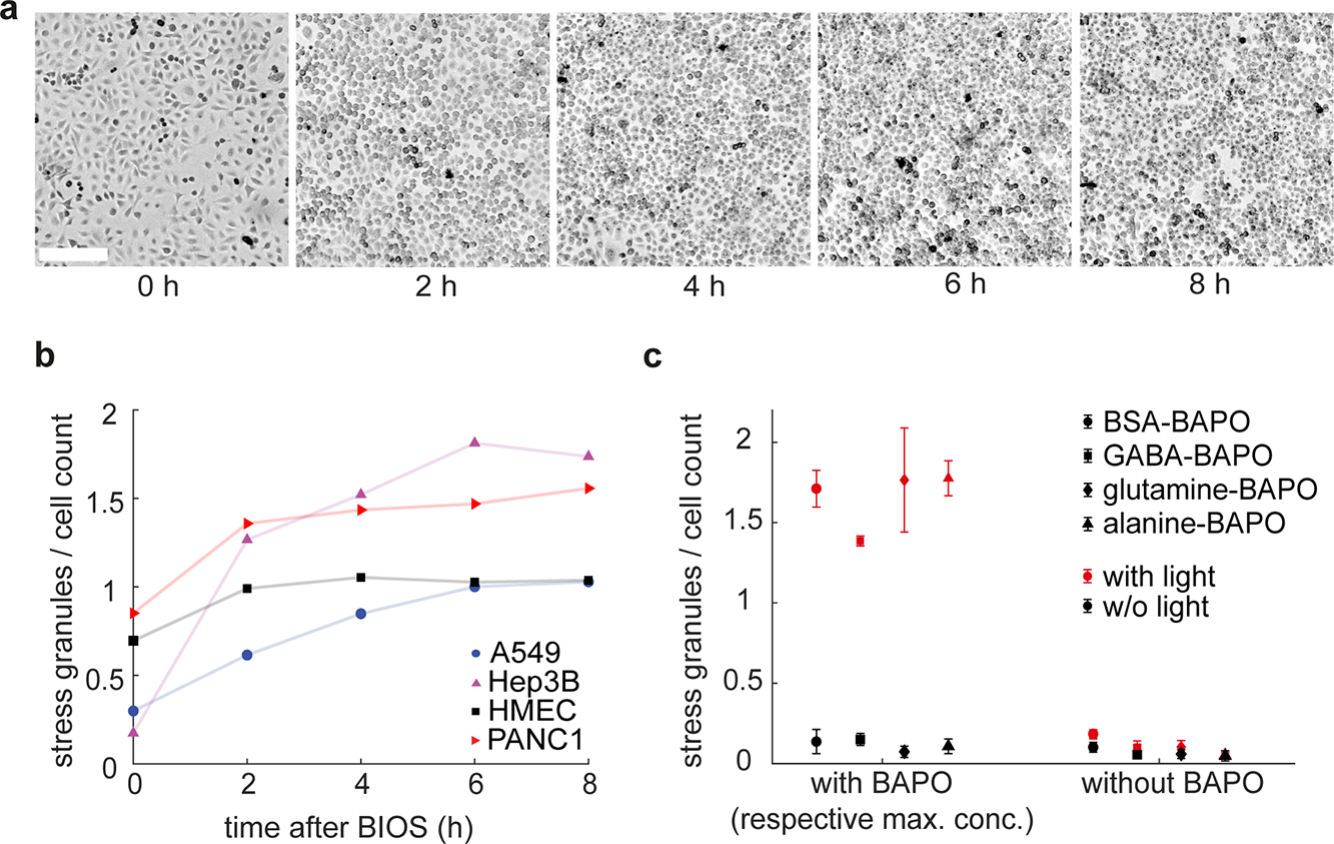
Timeline of cell death induction. (a) Representative bright field images of A549 cells acquired at the indicated times after BIOS induction. Scale bar: 200 μm. (b) Quantification of stress granule formation over time after BSA-BAPO treatment. (c) Quantification of stress granule formation 24 h after BIOS activation for all investigated BAPO compounds on A549 cells compared to controls. Condition “with BAPO” are treated at maximal concentrations investigated in the screen, i.e., 60/250/100/80 μM for BSA-/GABA-/glutamine-/alanine-BAPO, respectively. *N* = 3. Intensity for all experiments: 15 mW/cm^2^ for 30 min.

All library compounds induced cell death with high efficacy and exhibited negligible light/dark toxicity (Figure 2a,b for BSA-BAPO and Figure S10 for other compounds). When tracing cell morphology over time after BIOS induction, we observed hallmarks of controlled cell death (rounding and shrinking of cells, nuclear membrane disintegration, granule formation, loss of cell–cell contacts, and absence of membrane blebbing, Figure 3 and Figure S11).^27^ The prompt action of BIOS is emphasized by visible effects already detectable 2 h post-induction.

To further corroborate controlled cell death as the mode of action, we implemented a terminal deoxynucleotidyl transferase dUTP nick end labeling (TUNEL) assay. In agreement with the results from the CalA/EHD-based screens, the percentage of apoptotic cells detected by the TUNEL assay was significantly elevated 24 h after BIOS induction compared with control conditions (Figure S12).

Based on the previously studied photochemistry of BAPOs,^28^ we hypothesized that BIOS relies on the production of molecular radicals upon light irradiation. Radicals burden the cellular antioxidant system, resulting in radical-induced oxidative stress, which can ultimately trigger cell death.^29^ To confirm this hypothesis, we cotreated cells with 30 μM BSA-BAPO and different concentrations of the radical scavenger sodium ascorbate (Figure S13). Sodium ascorbate rescued all cell lines from BIOS, confirming radical-induced oxidative stress as a causative factor. The exception to this was the hepatocellular carcinoma-derived cell line Hep3B. This is attributable to the previously described toxicity of ascorbate to liver cancer,^30^ resulting from the combination of free transition metal ions and ascorbate, which leads to H_2_O_2_ production. Unlike the three other cell lines investigated, Hep3B cells exhibit suppressed glutathione peroxidase and catalase activities, which normally scavenge H_2_O_2_. Therefore, ascorbate alone is toxic to Hep3B cells.

### BIOS Enables Light-Controlled Cell Ablation *In Vivo*

While cell culture-based screens remain the most ethical, cost-effective, and time-effective method to assess the properties of a chemical agent in a biological system, they cannot capture the complexity of an organism. Driven by the positive outcomes of the cell-based screens, we moved to *in vivo* characterizations, employing the vertebrate species zebrafish (*Danio rerio*) at larval stages.

**Figure 4:**
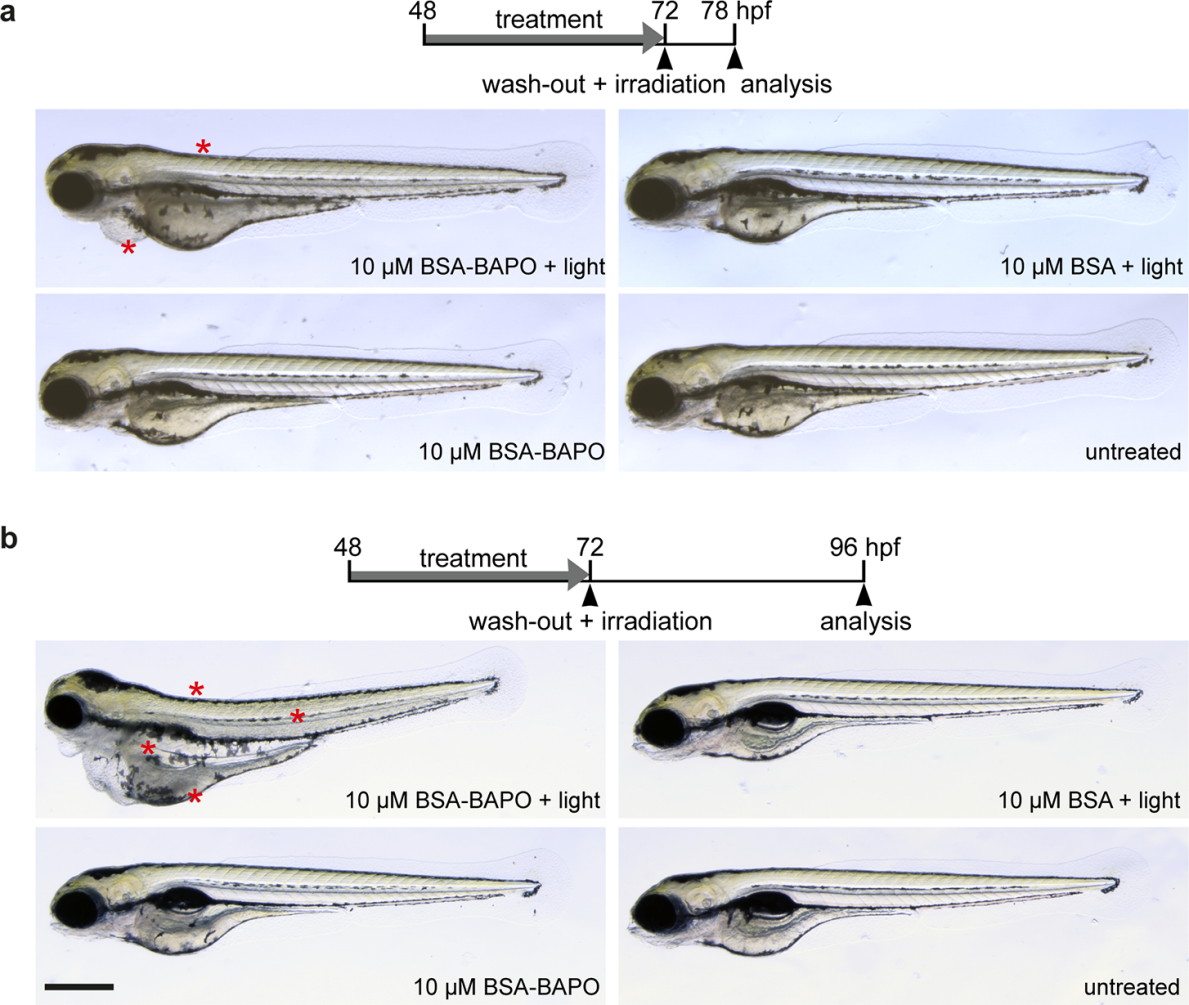
*In vivo* application of BIOS in zebrafish larvae. (a) Images of larvae acquired 6 h after BIOS activation. Loss of muscle tissue integrity and development of cardiac edema (asterisks) can be observed upon BIOS induction (upper left panel). Control conditions were unaffected (other panels). (b) Images of larvae acquired 24 h after BIOS activation. Exacerbated loss in muscle tissue integrity and cardiac edema (asterisks) were observed following BIOS treatment (upper left panel). No significant toxicity was observed for controlled conditions (other panels). Note that the control condition for irradiation only contains unmodified BSA to exclude any adverse effect of BSA alone. Scale bar: 500 μm for subfigure (a) and (b). Intensity: 15 mW/cm^2^ for 30 min.

First, we investigated the action of BAPOs at the organismic level. 48 h post-fertilization (hpf) larvae were treated with 10 μM BSA-BAPO, which was directly supplemented to the embryo media. At 72 hpf, BSA-BAPO was washed out followed by 30 min of light irradiation at 15 mW/cm^2^ for selected conditions. Larvae were microscopically investigated at 6 and 24 h post-irradiation (hpi). For larvae subjected to both BSA-BAPO and light irradiation, a systemic effect was observed, in particular, cardiac edema and loss of muscle tissue integrity, which emerged as early as 6 hpi (Figure 4a and Figure S14). At 24 hpi, the larvae subjected to BIOS developed severe cardiac edema (10/11) and severe loss of muscle tissue integrity (7/11, Figure 4b and Figure S15). This indicates that BSA-BAPO has a high bioavailability and was effectively distributed within the organism. All control conditions (untreated larvae, larvae subjected only to BSA-BAPO, and larvae subjected only to light irradiation in the presence of BSA, which was added to ensure no effect of BSA alone) did not show any signs of impaired conditions.

**Figure 5:**
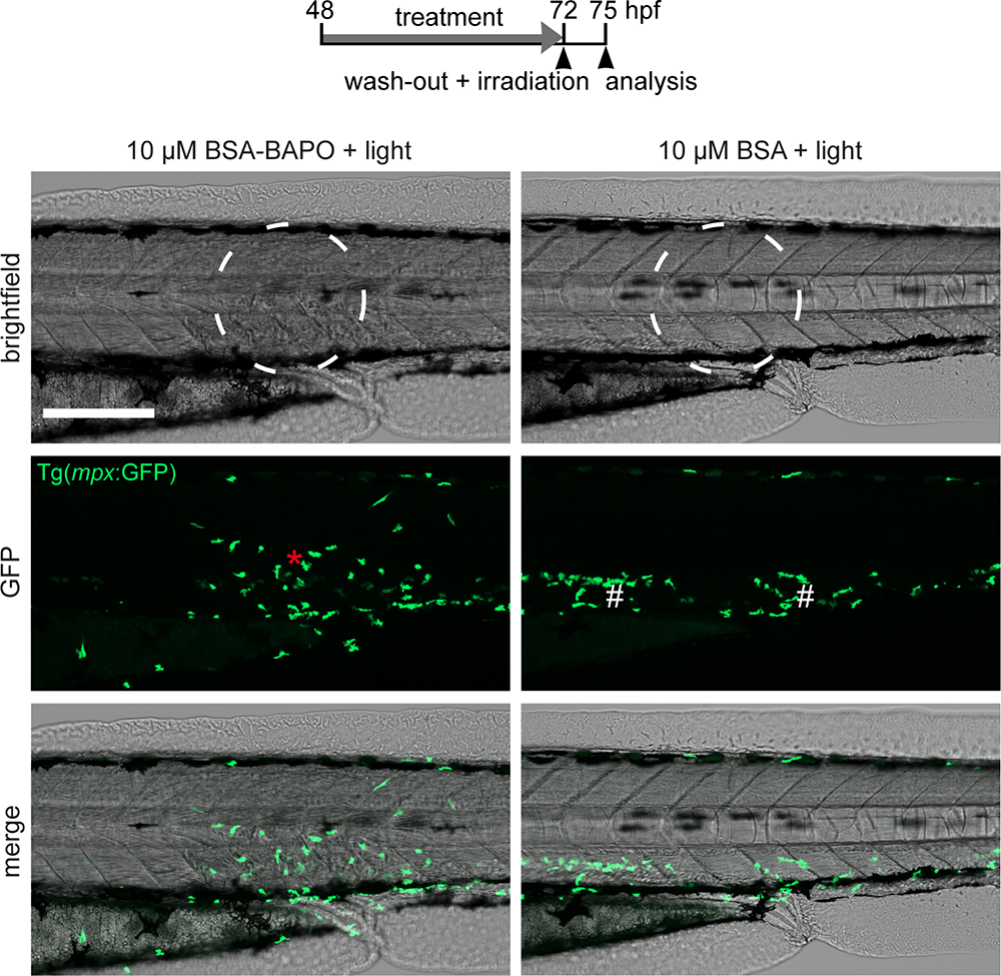
Local BIOS activation induces neutrophil recruitment in zebrafish larvae. Recruitment of neutrophils (green fluorescence, asterisk) following local BIOS (left column, irradiation area: white circle) in Tg(*mpx*:GFP) transgenic larvae at 3 hpi. Irradiation alone showed no effect on neutrophils (right column). White hash signs denote neutrophils in the caudal hematopoetic tissue, where they normally reside. Note the irradiation only control again contains unmodified BSA. Scale bar: 200 μm. Intensity: 9 mW/cm^2^ for 30 min.

Encouraged by this systemic effect, we then moved to local triggering of BIOS. Following the treatment protocol described above, we activated BSA-BAPOs locally in a circular area of approximately 250 μm in diameter in the trunk region at the level of the urogenital pore. The irradiated area showed clear signs of locally confined loss of tissue integrity, but only if the larvae were treated with BSA-BAPO and irradiated, while the light-only condition showed no detectable response (Figure 5 and Figure S16). Importantly, we employed the transgenic zebrafish line Tg(*mpx*:GFP), which enabled fluorescent detection of neutrophils, allowing for direct visualization of the tissue damage-induced immune response. The lesions in irradiated larvae treated with BSA-BAPO led to a rapid influx of neutrophils from the caudal hematopoetic tissue, where they normally reside, to the lesion site within 2 hpi (n = 3; Figure 5 and Figure S16),^31^ which demonstrates efficient BAPO-mediated cell ablation *in vivo*.

## Conclusions

We developed a platform to synthesize functionalized BAPOs, establishing a robust and customizable strategy to induce cell death *in vivo* and *in vitro* on demand. Our results have significant implications for fundamental research in chemical biology and biochemistry and, furthermore, have the potential for translation to the therapeutic space. Light-mediated spatiotemporal control enables local effect induction without harming neighboring cells. The target area is readily tuned via the illumination spot size. We verify that cell death induction is mediated by radical-induced oxidative stress, which immediately triggers immune cell influx to the target area in zebrafish larvae. In more therapy-related contexts, BAPOs exhibit promising characteristics: (i) Photosensitizers stay perennially active pending degradation or excretion, resulting in weeks-long side-effects after treatment.^32^ In contrast, BAPOs are activated once and are inactive before and afterward. This eliminates long-term side effects, e.g., after topical treatment. (ii) Radical formation in conventional photosensitizers is oxygen-dependent, which limits their applicability in hypoxic environments, which are commonly encountered in tumors. BAPO-mediated generation of radicals, in contrast, is oxygen-independent. (iii) The synthetic flexibility of the platform presented here enables straightforward tuning of the molecular properties of all components, specifically the phosphoryl and acyl parts of the BAPO, to the desired application. (iv) Advanced excitation schemes for BAPOs have been established and may further expand the application of BIOS *in vivo*. In particular, two-photon excitation-mediated cleavage of BAPOs has been reported.^33^ This shifts the required wavelength for activation into the near-infrared, improving penetration depth.^34^

Taken together, BAPOs demonstrate a versatile strategy to eliminate select cells *in vitro* and *in vivo*. Further optimization has the potential to find clinical applications where targeted induction of cell death is desired and novel approaches are urgently needed, such as in cancer, neurodegeneration, and infectious diseases.^35−37^

## Methods

### General Synthetic Techniques and Analytical Methods

All air- or moisture-sensitive reactions were carried out under dry argon by using either standard Schlenk techniques or working inside a glovebox (M-Braun Lab-Master MB 150 B-G). The glassware was stored in an oven at 130 °C for at least 16 h and cooled under a vacuum prior to use. Solvents were purified using an Innovative Technology PureSolv MD 7 solvent purification system and stocked over activated molecular sieves. When degassed solvents were used, nitrogen is bubbled for a minimum of 15 min. Deuterated solvents were purchased from Cambridge Isotope Laboratories. All reagents were used as received from commercial suppliers, unless otherwise stated. Air sensitive compounds were handled and stored in an M-Braun Lab-Master MB 150 B-G glovebox. Light sensitive compounds were handled with the exclusion of light. Dialysis was performed using Spectrum Spectra/Por 6 prewetted standard RC Dialysis tubing, 1–50kD MWCO. Lyophilization was performed using a Christ Epsilon 2–4–85 °C LSC plus Pilot Freeze-dryer. NHS-BAPO was synthesized as previously reported.^17,28^ NMR spectra were recorded on Bruker Avance 200, 250, 300, 400, and 500 spectrometers operating at RT if not otherwise specified. Chemical shifts δ were measured according to IUPAC and are given in parts per million (ppm) relative to TMS (1H-NMR and 13C-NMR) and 85% H3PO4 in D2O (31P-NMR). The multiplicity of the signals is indicated as s (singlet), d (doublet), t (triplet), q (quartet), or m (multiplet). The abbreviation “br” describes broad signals. Absolute values of coupling constants J are given in Hertz (Hz). Mass spectrometry experiments were carried out by the MS Service (MoBiAS, Laboratory of Organic Chemistry, D-CHAB, ETH Zürich). Elemental analyses were carried out by the Microlaboratory (Laboratory of Organic Chemistry, D-CHAB, ETH Zürich). For details on synthesis and characterization, see Figures S1–S4 and Supporting Information on spectroscopy.

### Cell Culture and Cell Seeding

A549, Hep3B, and PANC-1 cells were grown in Roswell Park Memorial Institute 1640 medium (RPMI) supplemented with 10% fetal bovine serum (FBS), 1% GlutaMAX (all Thermo Fisher), and 1% penicillin-streptomycin antibiotic cocktail (Sigma-Aldrich). HMECs were grown in MCDB 131 medium supplemented with 10% FBS, 1% GlutaMAX (all Thermo Fisher), and 1% Penicillin-streptomycin antibiotic cocktail (Sigma-Aldrich), 10 ng/mL hEGF (Thermo Fisher), and 1 μg/mL hydrocortisone (Sigma-Aldrich). All cells were cultivated in a humidified 5% CO_2_ atmosphere at 37 °C until approximately 80% confluency was reached. Cells were subcultured twice a week. Confluent cells were washed with 5 mL of phosphate buffer saline (PBS) without Ca^2+^/Mg^2+^ (Thermo Fisher) and detached with 1 mL trypsin-EDTA (Thermo Fischer). Cells were seeded into flat-bottom 96-well plates (Corning) before BAPO incubation and irradiation.

### *In Vitro* Screens

When cell lines approached confluence, BAPOs were added at the respective concentrations. Untreated control wells were incubated with the respective amount of solvent used for the corresponding BAPO preparation (media for BSA-BAPO and DMSO for the other BAPOs, maximum DMSO concentration below 1%). The plates were incubated for 1 day to allow for cellular uptake. Thereafter, the cells were washed with a cell culture medium to ensure that the observed effect on cells was a result of the internalized BAPO rather than the excess in the media. BIOS was induced via irradiation with 365 nm light at 15 mW/cm^2^ for 30 min.

### Irradiation

Irradiation was usually performed using the custom irradiation device described in Figure S6. For fiber-based irradiation experiments, the custom setup described in Figure S7 was used. For local irradiation (Figures 1d and 5, and Figures S5 and S16), the emission of a mercury lamp passed through a 365/10 nm bandpass filter (Chroma) was focused onto the target area (approximately 240 μm in diameter, intensity of 9 mW/cm^2^) using a C-Apochromat 40*x*/1.2 W Korr UV–vis-IR objective (Zeiss) installed on a commercial confocal microscopy setup (LSM980, Zeiss).

### Viability Assays

Cells were incubated with 4 μM calcein AM (CalA, Corning) and 2 μM ethidium homodimer-1 (EHD, Sigma-Aldrich) in media. The assay relies on detecting the loss of membrane integrity and esterase activity as a measure of cell viability. In alive, metabolizing cells, nonfluorescent CalA is rapidly converted to fluorescent calcein by cytosolic esterases. CalA fluorescence indicates live cells. On the other hand, EHD cannot penetrate the membrane of alive cells. If the plasma and nuclear membranes are compromised during cell death, EHD enters the cell and intercalates into DNA. Thus, nuclear fluorescence from EHD indicates dead/dying cells. After approximately 10 min, images of the center of the wells (approximately 1.7 × 1.7 mm^2^) were acquired utilizing a plate reader (ImageXpress Pico, Molecular Devices). The detected fluorescent signal was analyzed by using Fiji. CalA and EHD fluorescence was analyzed by utilizing the same approach. An appropriate threshold was applied to all images in the stack. To account for uneven fluorescence signals, heterogeneous cell size, and confluency of cells, binary transformation, appropriate particle size threshold, and the Watershed algorithm were applied to the analysis, respectively. Parameters were kept identical for identical cell lines and channels within one experiment. Viability was calculated as the ratio of live cells to the total number of cells. Cells that showed both CalA and EHD fluorescence, which were occasionally observed, were counted as dead.

### TUNEL Assay

To investigate apoptosis, a TUNEL assay was performed using the Click-iT Plus TUNEL Assay for in situ apoptosis detection, Alexa Fluor 647 dye (Invitrogen) as per the manufacturer’s protocol. A549 cells were treated with 60 μM alanine BAPO for 24 h, washed, and then irradiated with 365 nm light at 15 mW/cm^2^ for 30 min as described above. One day after irradiation, the cells were fixed with 4% paraformaldehyde solution (Thermo Fisher) in PBS for 15 min at RT. After washing the fixative agent with PBS, cells were permeabilized using 0.25% Triton X-100 in PBS at RT followed by another washing step with PBS. Cells were then incubated with terminal deoxynucleotidyl transferase (TdT) reaction buffer at 37 °C for 10 min followed by incubation in TdT reaction mixture at 37 °C for 1 h. After washing with 3% (w/v) BSA in PBS for 2 min, the cells were incubated with a freshly made Click-iT Plus TUNEL reaction cocktail at 37 °C for 30 min followed by a final wash in 3% (w/v) BSA in PBS for 5 min. To identify nonapoptotic cells, the cells were incubated with 1 μg/mL of Hoechst 33342 for 20 min at RT and then washed with PBS.

### Morphological Analysis

Bright-field images were acquired directly after BIOS induction as well as 2, 6, and 8 h after. Stress granules were quantified by using appropriate intensity and particle size thresholds. Granule counts were then normalized by the number of cells in the field of view. Initially, the total number of cells in the field of view is quantified via setting an appropriate threshold that separates the background from cell outlines. This parameter choice was robust under all conditions. A watershed algorithm was used to separate the touching cells. Particle counting was applied to count the number of cells in the field of FOV, setting a limit for the particle size to exclude occasionally detected objects that are too small to be cells. Stress granules are visibly darker spots within the cells in the bright-field images. Thus, they can be easily identified via thresholding. Due to their narrow size distribution, it is straightforward to count them via particle counting in combination with an upper limit for the maximal size, again excluding occasionally detected objects that are too large to be granules. We added this information to the manuscript. For example images, please see SI Figure 11.

### Rescue Experiments

Cells were cotreated with 30 μM BSA BAPO and a respective concentration of sodium ascorbate. Irradiation, cell viability assay, imaging, and image analysis were performed as described above.

### Tissue Phantoms and Fiber-Based Irradiation

To investigate the optical effects tissue exhibits on BIOS activation (e.g., scattering and absorption), tissue-mimicking phantoms were prepared as described before.^24^ Briefly, 2 g of bacteriological agar (VWR) and 0.05 g of aluminum oxide (Thermo Fisher) were dissolved in 55 mL of water supplemented with 25 μL of black ink (Pelikan). The mixture was then heated to 94 °C, poured into rectangular aluminum molds (3 mm depth), and left to cool at RT. The mold was then cooled to 4 °C for 24 h in order to solidify. The next day, cells were treated using 80 μM alanine BAPO and were covered by phantoms or not. To mimic fiber-based irradiation as required for activation in tissue, a custom setup was used (see Figure S7 for details). The power at the sample was the same as that for the previous experiments. The cell viability assay, imaging, and image analysis were performed as described above.

### In Vivo Zebrafish Larvae Screens

Two days postfertilization (dpf), zebrafish larvae (wild type AB strain) were treated with either 10 μM BSA-BAPO or 10 μM BSA directly added to the water. After 1 d, the larvae were washed thoroughly, anesthetized in E3 medium containing 0.02% MS-222 (PharmaQ, Tricaine PharmaQ), and irradiated using the irradiation device (15 mW/cm^2^ for 30 min). For local irradiation experiments, we used Tg(*mpx*:GFP)^uvm1^ transgenic zebrafish expressing GFP under regulatory elements of the *mpx* gene to label neutrophils.^38^ BSA/BSA-BAPO incubation was performed as described above. For irradiation, larvae were anesthetized in 0.02% MS-222 and mounted in a lateral position in 1% low melting point agarose (Ultra-PureTM Low Melting Point, Invitrogen 16520) between two microscope cover glasses. During irradiation, larvae were covered with 0.01% MS-222-containing E3 medium to keep the preparations from drying out. Irradiation was performed by passing filtered light from a mercury lamp through a C-Apochromat 40*x*/1.2 W Korr UV–vis-IR40x objective (Zeiss) installed on a commercial confocal microscopy setup (LSM980, Zeiss). Fluorescent images were acquired using a Plan-Apochromat 10*x*/0.45 M27 objective installed on a laser scanning confocal microscope (LSM980, Zeiss). Brightfield overviews were acquired using a Leica M205 FCA stereo microscope equipped with a Leica DMC6200 C color camera.

### Statistics

Due to the multitude of conditions to be compared, we do not show significance levels via symbols in the respective plots. All statistical analyses are summarized in a comprehensive Excel sheet, available as additional information. Graphs show mean ±SD. As three or more groups were compared in every experiment, a one-way ANOVA followed by Bonferroni correction was performed. Pairwise *t* tests were performed for TUNEL assay data sets to evaluate the significance.

### Software

OriginPro 8G was used for data visualization, analysis, and statistics. Images were analyzed by using ImageJ. Figures were compiled in Adobe Illustrator.

## Data Availability

All raw images are available at figshare (https://figshare.com/s/9295a731ad6315536af9). Other inquiries can be directed to LM.

## Supporting Information Available

BAPO statistics (XLSX)
The supplementary document provides detailed characterization of BAPO conjugate synthesis, irradiation devices and setups, biological and irradiation screens, tissue phantom experiments, and zebrafish larvae studies (PDF)
