# Supplementary material for: Tailored Bisacylphosphane Oxides for Precise Induction of Oxidative Stress-Mediated Cell Death in Biological Systems
Source: ACS Chem Biol. 2024 Nov 5;20(1):77–85. doi: 10.1021/acschembio.4c00399 (PMC11744658; doi:10.1021/acschembio.4c00399)
Supplement: Supplementary file 2 — cb4c00399_si_002.pdf [file cb4c00399_si_002.pdf]

Electronic Supplementary Information for

Tailored Bisacylphosphane Oxides for precise induction of oxidative stress-mediated cell death in biological systems

Karim Almahayni, Jana Bachir Salvador, Riccardo Conti, Anna Widera, Malte Spiekermann, Daniel Wehner, Hansjörg Grützmacher, and Leonhard Möckl

Table of contents

- Figure S1: Glutamine-BAPO characterization.
- Figure S2: Alanine-BAPO characterization.
- Figure S3: GABA-BAPO characterization.
- Figure S4: BSA-BAPO characterization.
- Figure S5: Local BIOS activation in A549 cells.
- Figure S6: Irradiation device.
- Figure S7: Fiber-based irradiation device.
- Figure S8: Irradiation screens.
- Figure S9: Irradiation in the presence of tissue phantoms.
- Figure S10: BAPO conjugates screens.
- Figure S11: Changes in cell morphology over time following BIOS.
- Figure S12: TUNEL assay
- Figure S13: Rescue upon ascorbate treatment.
- Figure S14: Zebrafish larvae 6 h after BIOS induction.
- Figure S15: Zebrafish larvae 24 h after BIOS induction.
- Figure S16: Local induction of BIOS in zebrafish larvae triggers neutrophil recruitment
- Supplementary information on spectroscopic BAPO characterization and BSA-BAPO synthesis

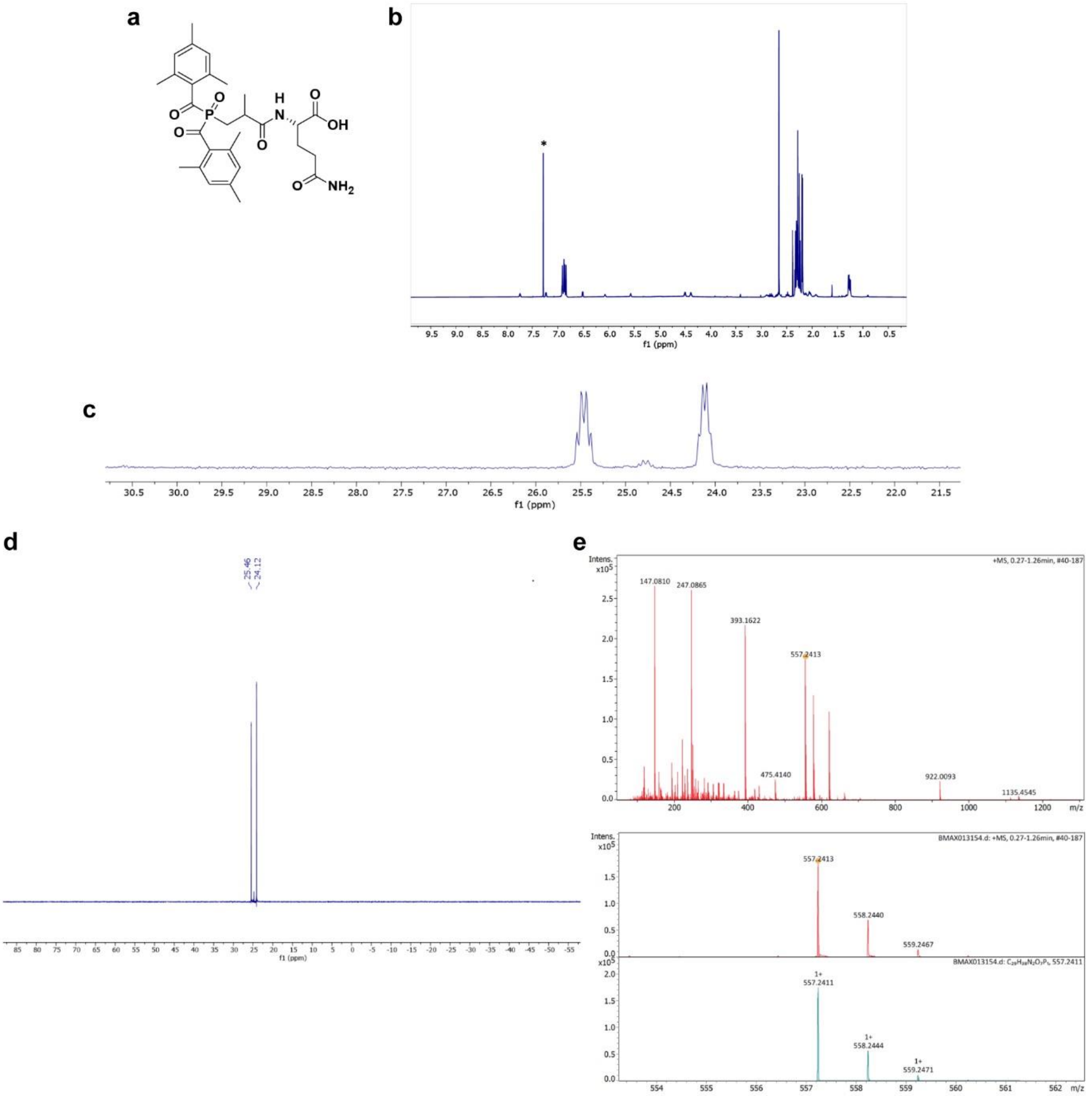

Figure S1. Glutamine-BAPO characterization.
Prepared according to the general ligation procedure. Yield: 57% (180 mg). Yellow, hygroscopic solid. (a) Structure. (b) ^{1}H NMR (500 MHz, $CDCl_3$). (c) ^{31}P NMR (203 MHz, $CDCl_3$). (d) ^{31}P{1H} NMR (203 MHz, $CDCl_3$). (e) ESI-TOF (m/z) (+). See supplementary information on spectroscopic BAPO characterization for further details at the end of this document.

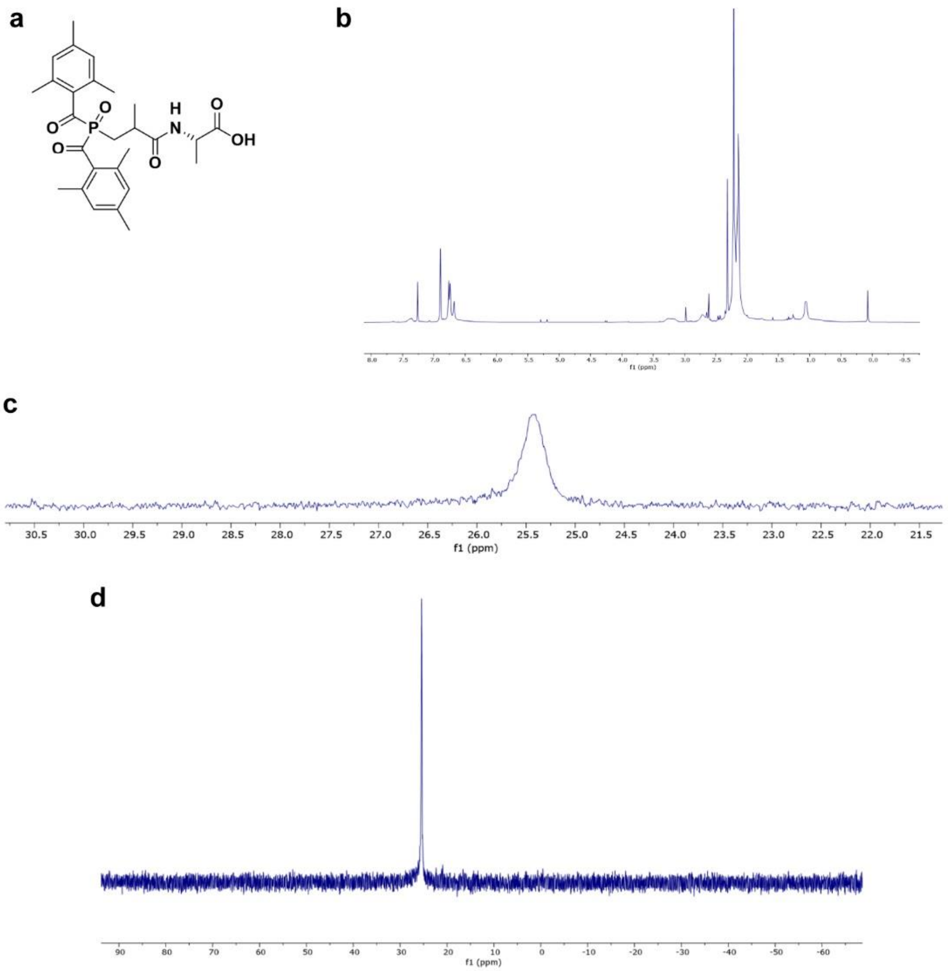

Figure S2. Alanine-BAPO characterization.
Prepared according to the general ligation procedure. Yellow, hygroscopic solid.
(a) Structure. (b) ^{1}H NMR (300 MHz, $CDCl_3$). (c) $^{31}P\{1H\}$ NMR (121 MHz, $CDCl_3$). (d) ^{31}P NMR (121 MHz, $CDCl_3$). See supplementary information on spectroscopic BAPO characterization for further details at the end of this document.

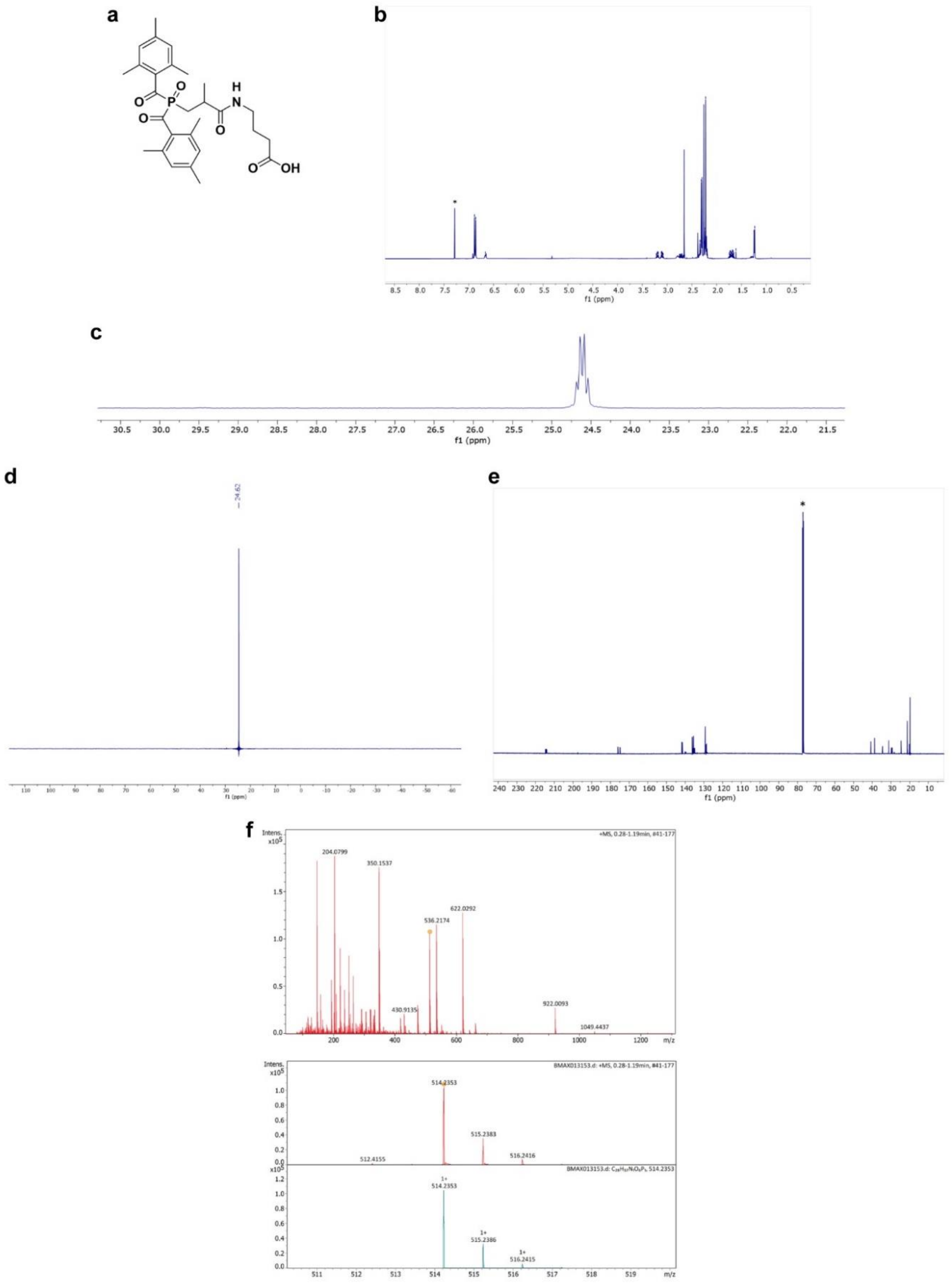

Figure S3. GABA-BAPO characterization.

Prepared according to the general ligation procedure. Yield: 78% (230 mg). Yellow, hygroscopic solid. (a) Structure. (b) ^{1}H NMR (500 MHz, $CDCl_3$). (c) ^{31}P NMR (203 MHz, $CDCl_3$). (d) ^{31}P{1H} NMR (203 MHz, $CDCl_3$). (e) ^{13}C{1H} NMR (126 MHz, $CDCl_3$). (f) ESI-TOF (m/z) (+). See supplementary information on spectroscopic BAPO characterization for further details at the end of this document.

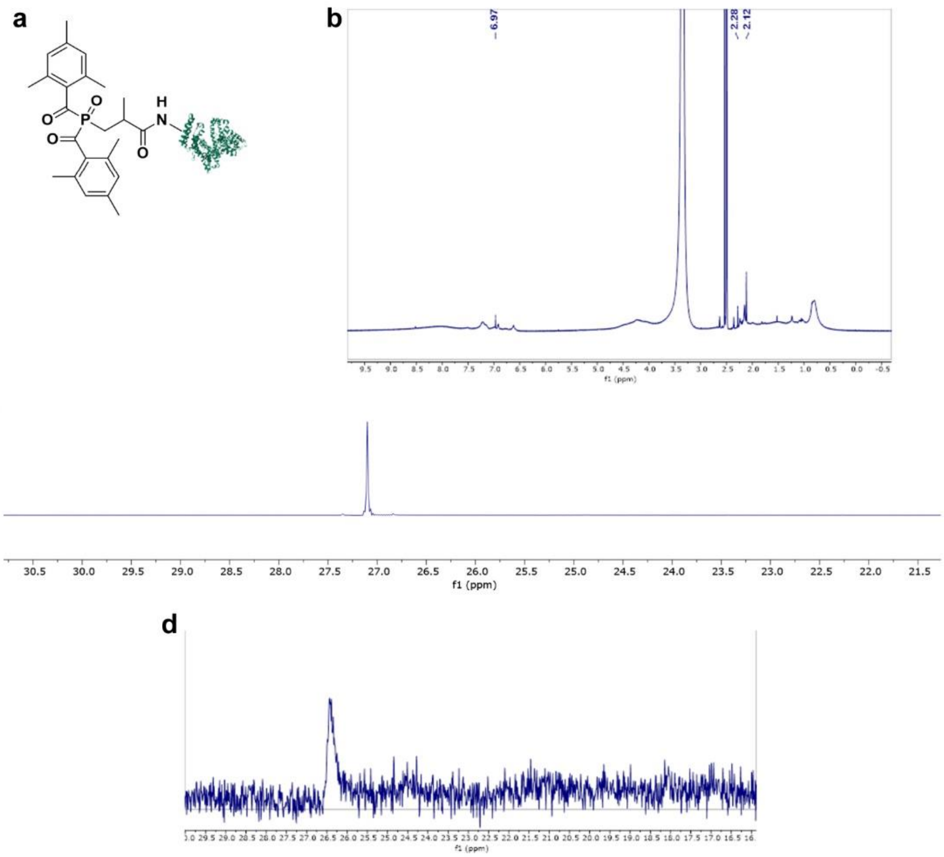

Figure S4. BSA-BAPO characterization.
(a) Structure. (b) ^{1}H-NMR (300.1 MHz, DMSO-d6, 298 K). (c) $^{31}P\{^{1}H\}$-NMR (121.5 MHz, DMSO-d6, 298 K). (d) ^{31}P-NMR (121.5 MHz, DMSO-d6, 298 K). See supplementary information on spectroscopic BAPO characterization for further details at the end of this document.

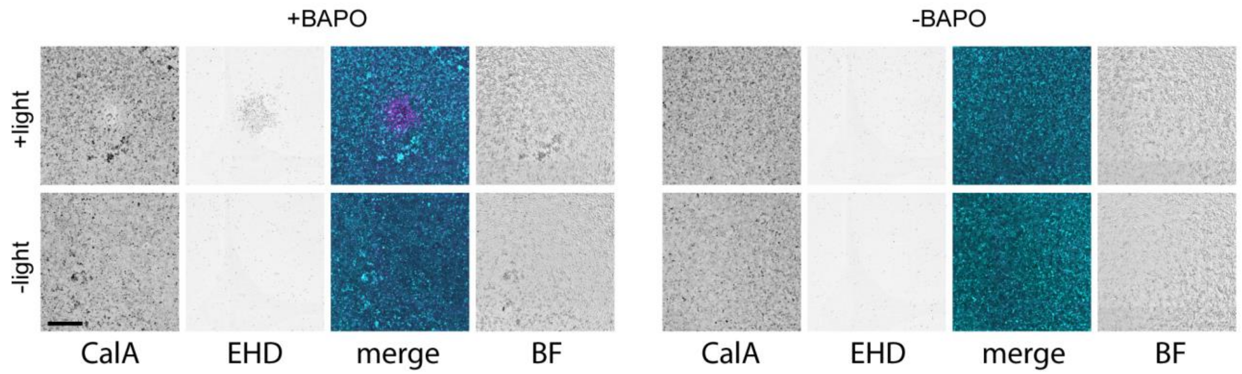

Figure S5. Local BIOS activation in A549 cells (alanine-BAPO, 60 μM). Local induction of cell death following BIOS (upper left). BIOS exhibits no dark toxicity (lower left) or light toxicity (upper right) compared to the untreated and unirradiated control (lower right). Scale bar: 500 μm. First image (left to right): Calcein A stain, second image: ethidium homodimer stain, third image: merge of CalA and EHD channels (CalA: cyan, EHD: magenta), fourth image: bright field image. Inverted LUT for single-channel fluorescence images (black corresponds to high signal). Light was delivered via a 40x Objective (see Methods).

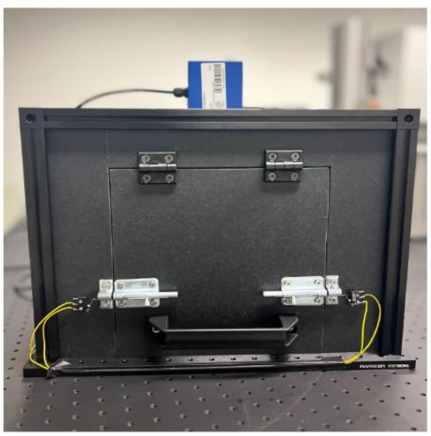

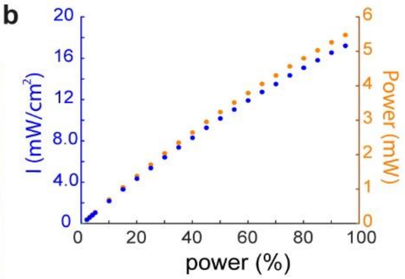

c

0.74	0.85	0.92	0.91	0.85	0.75
0.82	0.92	0.97	0.98	0.91	0.84
0.82	0.94	0.98	1.00	0.94	0.83
0.77	0.91	0.97	0.97	0.93	0.80
0.65	0.84	0.88	0.89	0.84	0.72

Figure S6. Irradiation device. (a) Photograph of the irradiation device. (b) Measured intensity in dependence on the power setting. The sensor was placed in the location where cells are irradiated. (c) Intensity variation over the irradiated area. Values are normalized, and measurement locations are spaced 2.5 cm horizontally and vertically from each other. The yellow rectangle denotes the area of the 96-well plates that contained cells in our experiments. Homogenous irradiation for all cells is ensured.

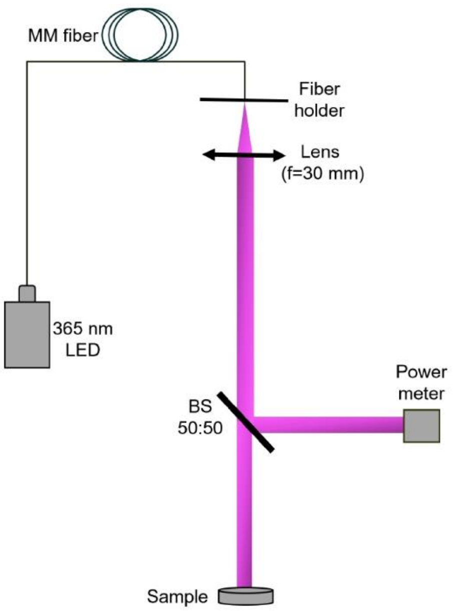

Figure S7. Fiber-based irradiation device. A fiber-coupled light emitting diode (LED, Thorlabs M365FP1) was used. The LED was connected to a LED driver (Thorlabs LEDD1B) at continuous wave mode and coupled into a multi-mode (MM) 600 µm core diameter fiber, 0.22 NA (Thorlabs M143L01). Light emitted from the fiber was collected by a lens (f=30 mm, Thorlabs LA1805) placed 3 cm away from the fiber holder opening, and directed downwards. A 50/50 beam splitter (BSW19, Thorlabs) was placed 15 cm away from the lens, splitting the light into two arms. One arm was directed through the tissue phantom onto the sample, and one arm was directed to a power meter (Thorlabs PM100D) to precisely measure the delivered power.

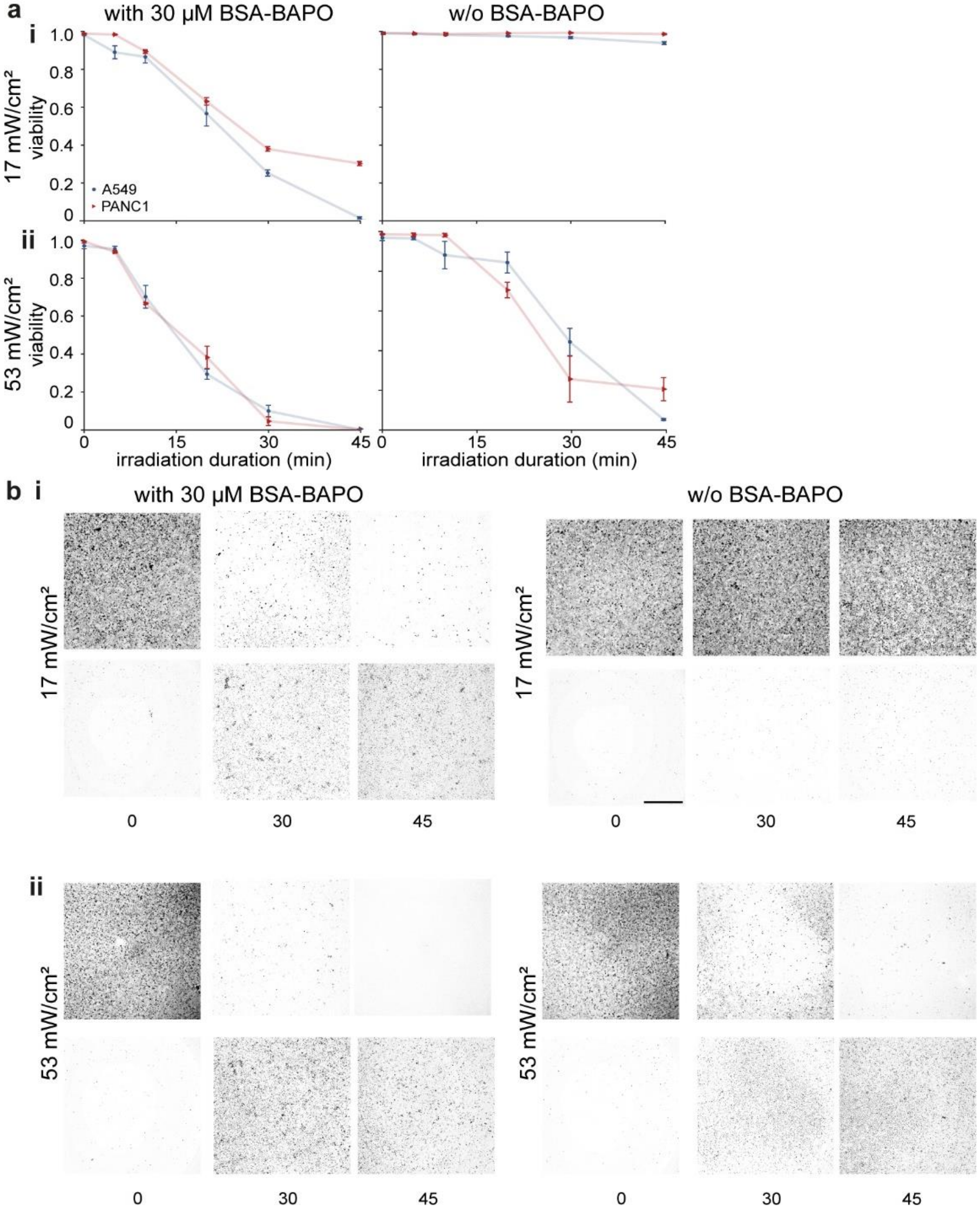

Figure S8. Irradiation screens. (a) Quantification of cell viability of A549 (blue) and PANC-1 (red) cells when treated with light doses of either (i) 17 mW/cm^2 or (ii) 53 mW/cm^2 for 45 minutes in presence of 30 µM BSA-BAPO or without (left and right panels, respectively). N=3. Error bars: SD. (b) Representative images of A549 cells when treated with light doses of either (i) 17 mW/cm^2 or (ii) 53 mW/cm^2 for 45 minutes with 30 µM BSA-BAPO or without (left and right, respectively). Upper row: Calcein stain (live cells); bottom row: ethidium homodimer stain (dead cells). Inverted LUT. Scale bar: 1 mm.

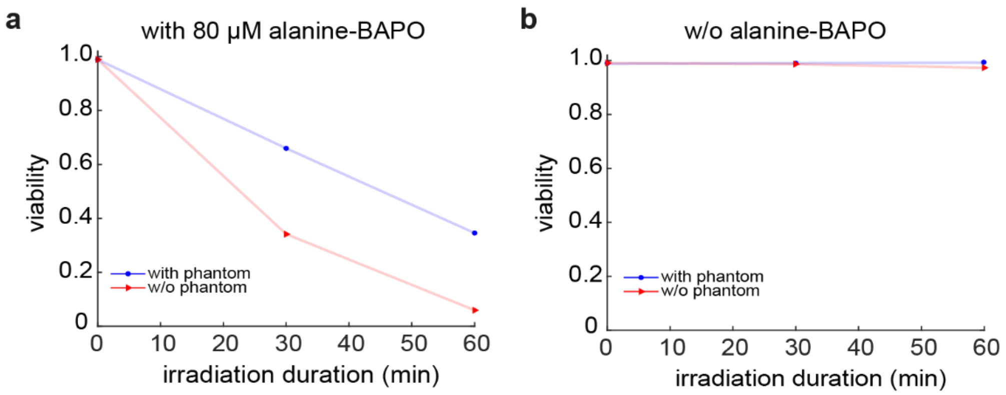

Figure S9. Irradiation in the presence of tissue phantoms. Quantification of cell viability of A549 when treated with a light dose of 15 mW/cm^2 with (a) 80 μM alanine-BAPO or (b) without in the presence or absence of a tissue phantom (blue and red, respectively).

a

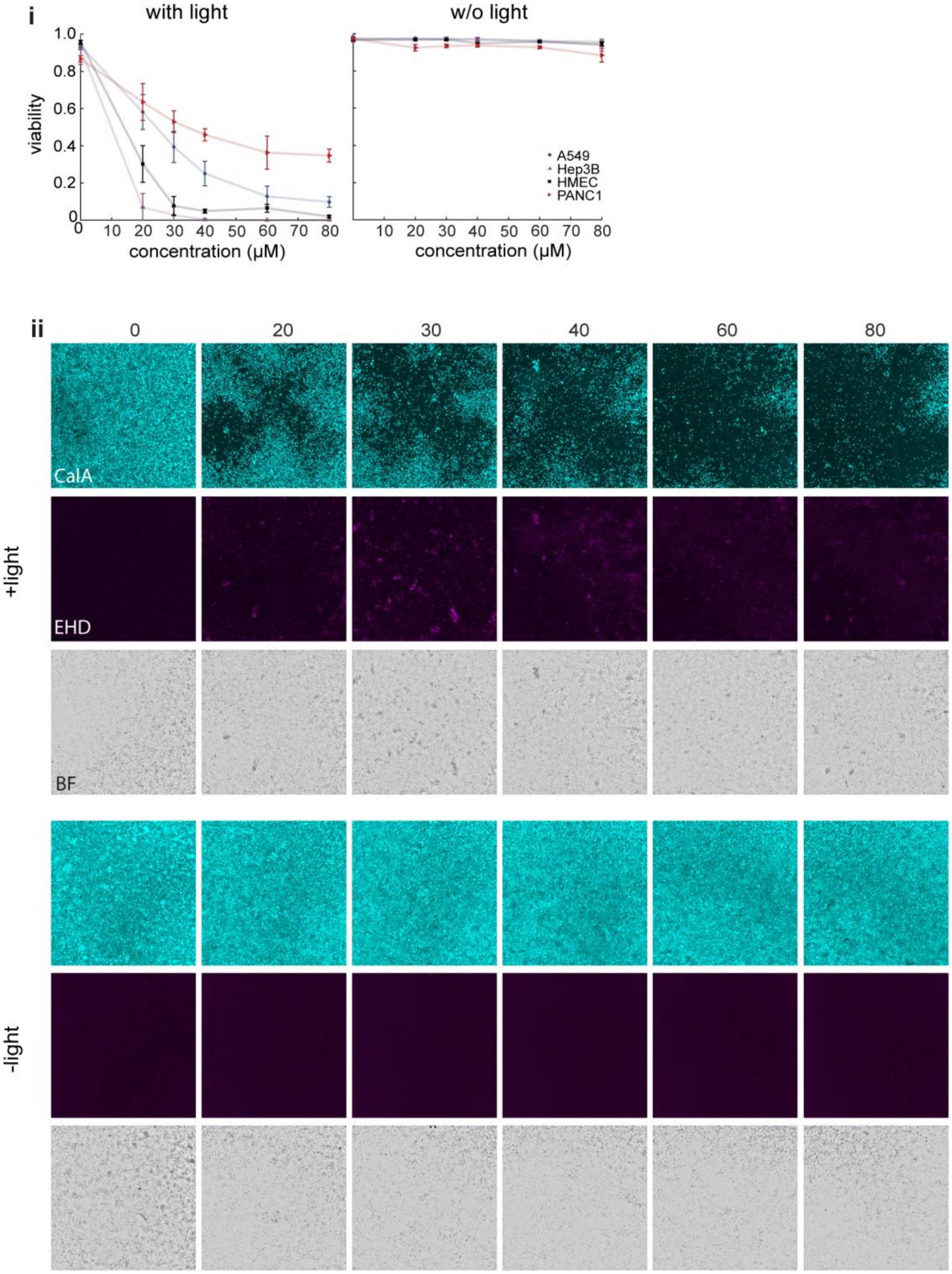

i
with light
w/o light
viability
1.0
0.8
0.6
0.4
0.2
0
0 10 20 30 40 50 60 70 80
concentration (µM)
10 20 30 40 50 60 70 80
concentration (µM)
A549
Hep3B
HMEC
PANC1
ii
0
20
30
40
60
80
CalA
EHD
BF
+light
-light

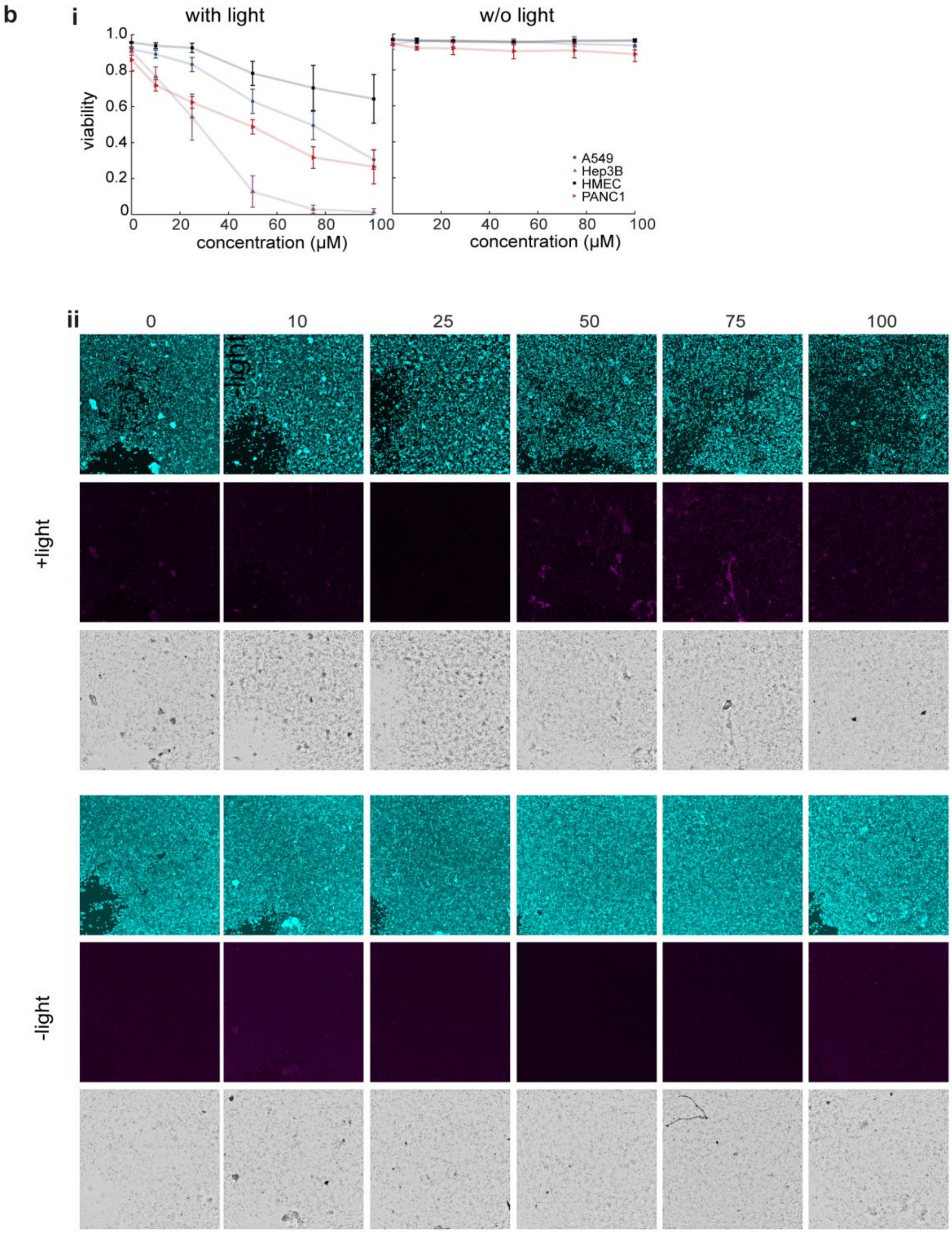
b
i
with light
w/o light
viability
1.0
0.8
0.6
0.4
0.2
0
0
20
40
60
80
100
concentration (µM)
A549
Hep3B
HMEC
PANC1
ii
0
10
25
50
75
100
+light
-light

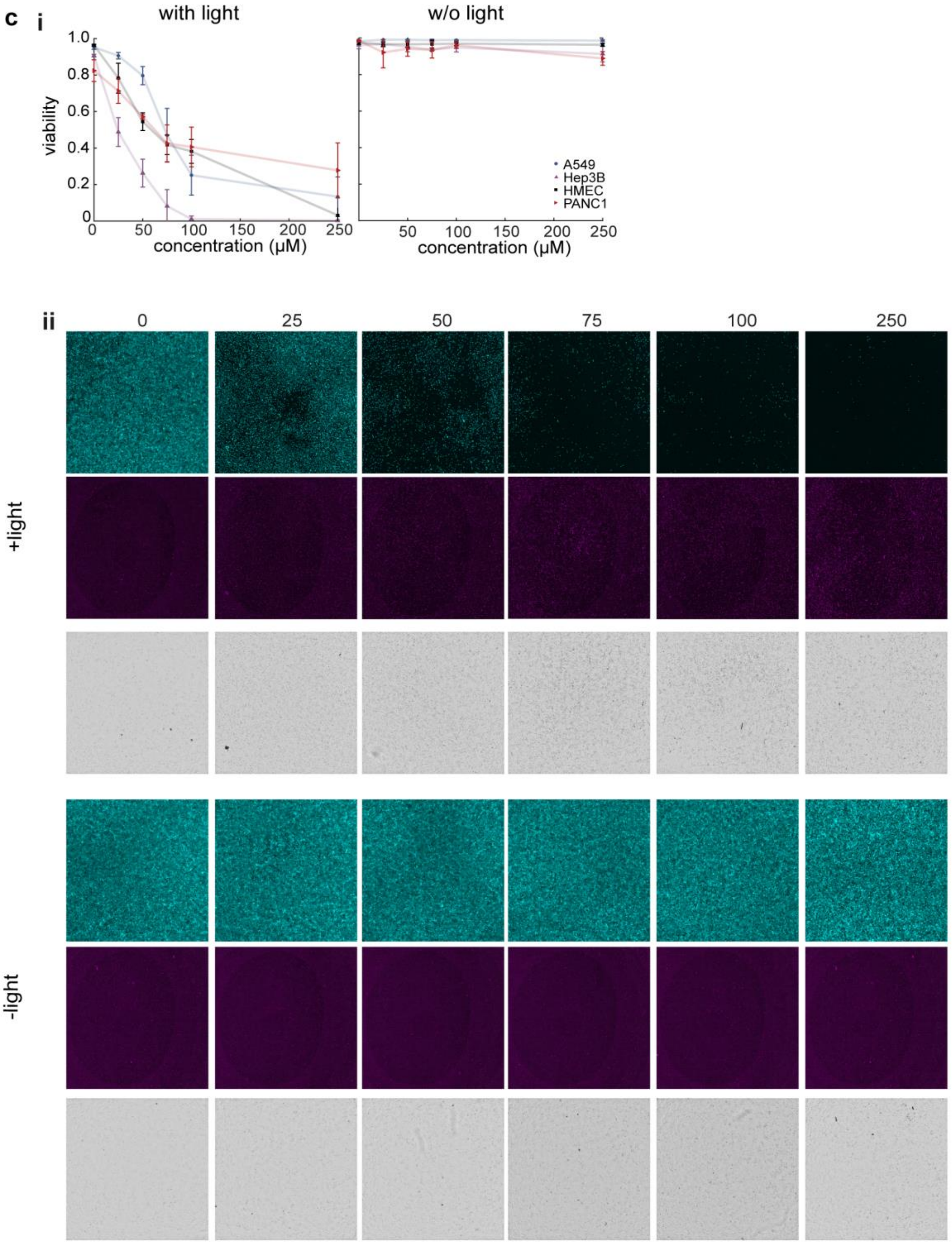

Figure S10. BAPO conjugates screen. (i) Quantification of viability of cells treated with (a) alanine-BAPO, (b) glutamine-BAPO, or (c) GABA-BAPO and either subjected to light irradiation or not (left/right panel, respectively). N=3. Error bars: SD. (ii) Representative images of A549 cells, PANC-1 cells, HMECs, respectively. Upper row: CalA stain (live cells; cyan); bottom row: EHD stain (dead cells; magenta). Irradiation intensity: 15 mW/cm^2. Scale bar: 500 µm

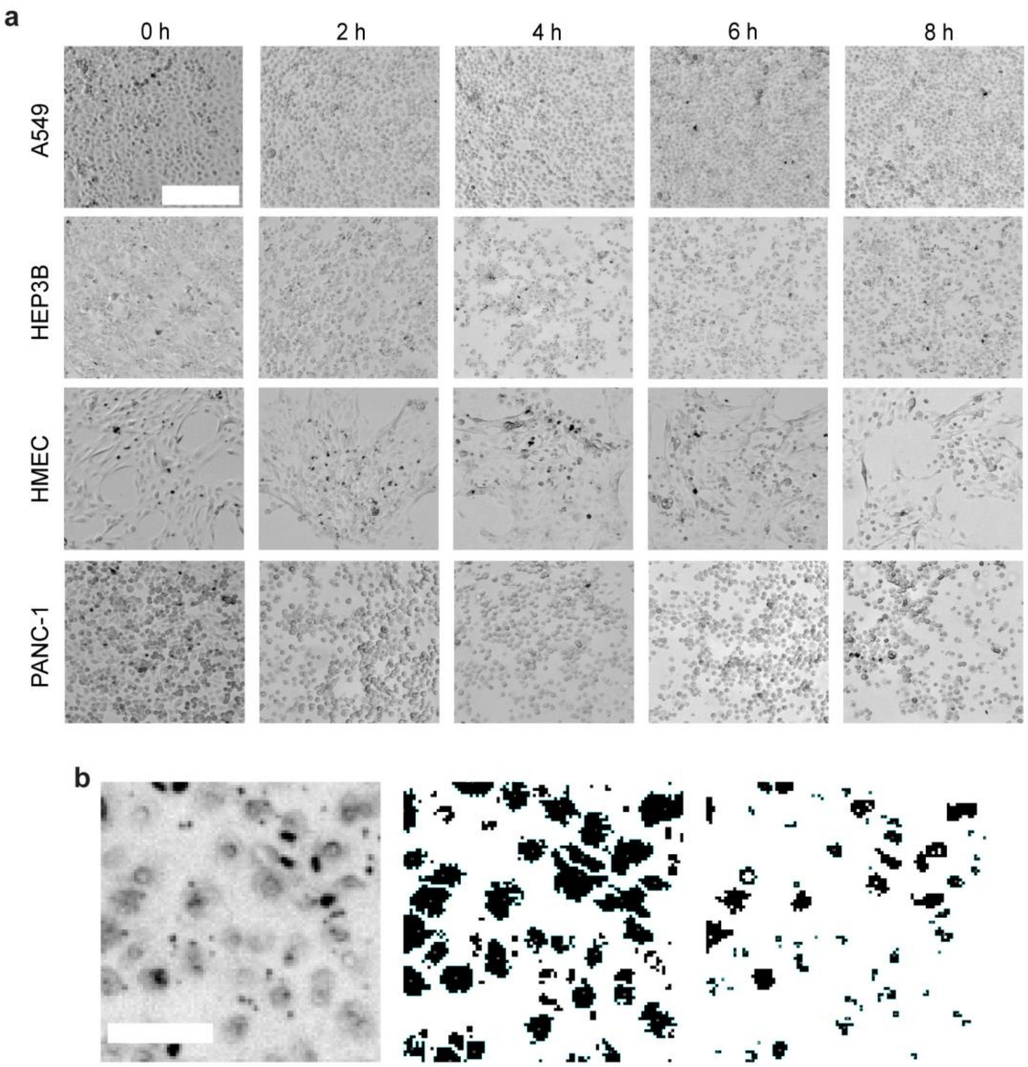

Figure S11. Changes in cell morphology over time following BIOS and stress granule production. (a) Representative images of A549 cells, Hep3B cells, HMECs, PANC-1 cells (top to bottom) 0 to 8 hours after BIOS induction (left to right). The change in morphology is demonstrated here across four different cell lines, once for each cell line. Scale bar: 500 µm. (b) Example area of a FOV at a zoom level were single cells and stress granules are visible. Left: Bright field. Middle: Cell outlines (cyan). Right: Stress granules counted (cyan outlines). Scale bar: 50 µm.

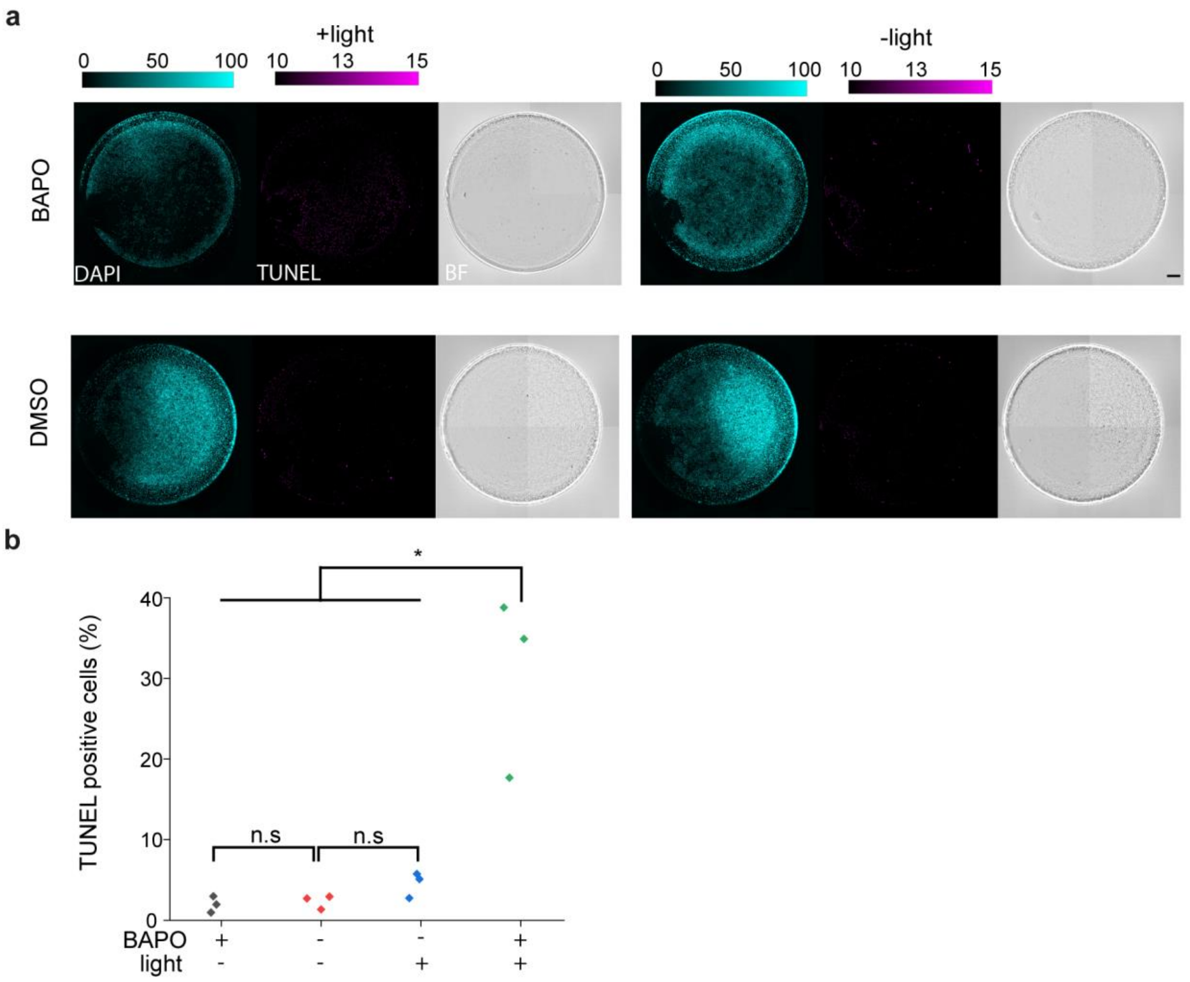

Figure S12. TUNEL assay. (a) Representative images of A549 cells stained for TUNEL-positive cells (right panel) and with DAPI (left panel). For cells subjected to BIOS, elevated TUNEL signal was observed (upper right) compared to control conditions (other panels). (b) Quantification of TUNEL positive cells. Scale bar: 500 µm. Pairwise t-tests were performed for the datasets to evaluate the significance, * indicates $p \leq 0.05$. Note that the FOV was imaged with a higher magnification objective, which requires four images to capture the full FOV in a 2x2 grid. The different objective position in relation to the wall of the well causes slightly different illumination, resulting in small differences in brightness.

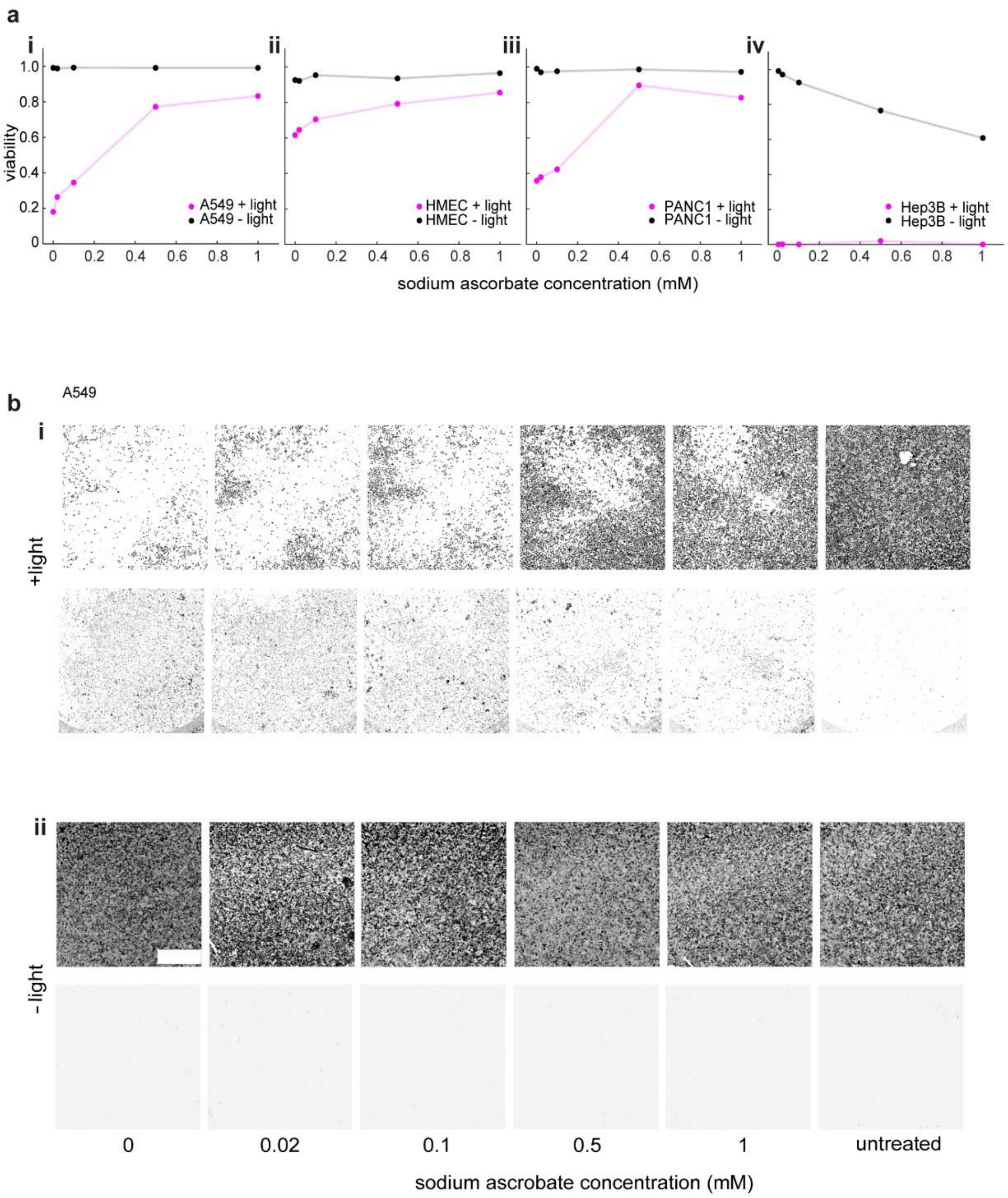

Figure S13. Rescue upon ascorbate treatment. (a) Quantification of cellular viability of (i) A549 cells, (ii) HMECs, (iii) PANC-1 cells, (iv) Hep3B cells when co-treated with 30 μM of BSA-BAPO and ascorbate at the indicated concentrations and either subjected to light irradiation or not (black, purple symbols, respectively). (b) Representative images of A549 cells when co-treated with 30 μM of BSA-BAPO and different ascorbate concentration as indicated. (i) corresponds to light irradiation, (ii) to no light irradiation. Upper rows: CalA stain (live cells); bottom row: EHD stain (dead cells). Untreated refers to no treatment with neither BSA-BAPO nor ascorbate. Inverted LUT. Scale bar: 1 mm.

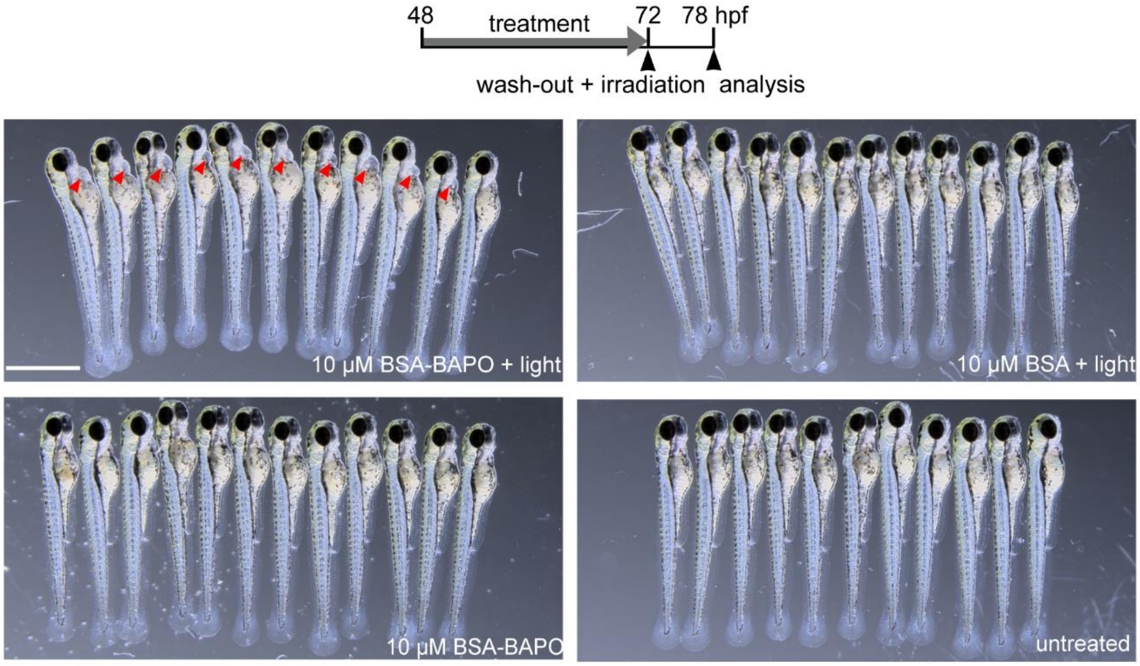

Figure S14. Zebrafish larvae 6 h after BIOS induction. All larvae investigated are shown. Loss of tissue integrity and development of cardiac edema (red arrowheads) are visible for larvae subjected to BIOS. Control conditions are unaffected. Scale bar: 1 mm.

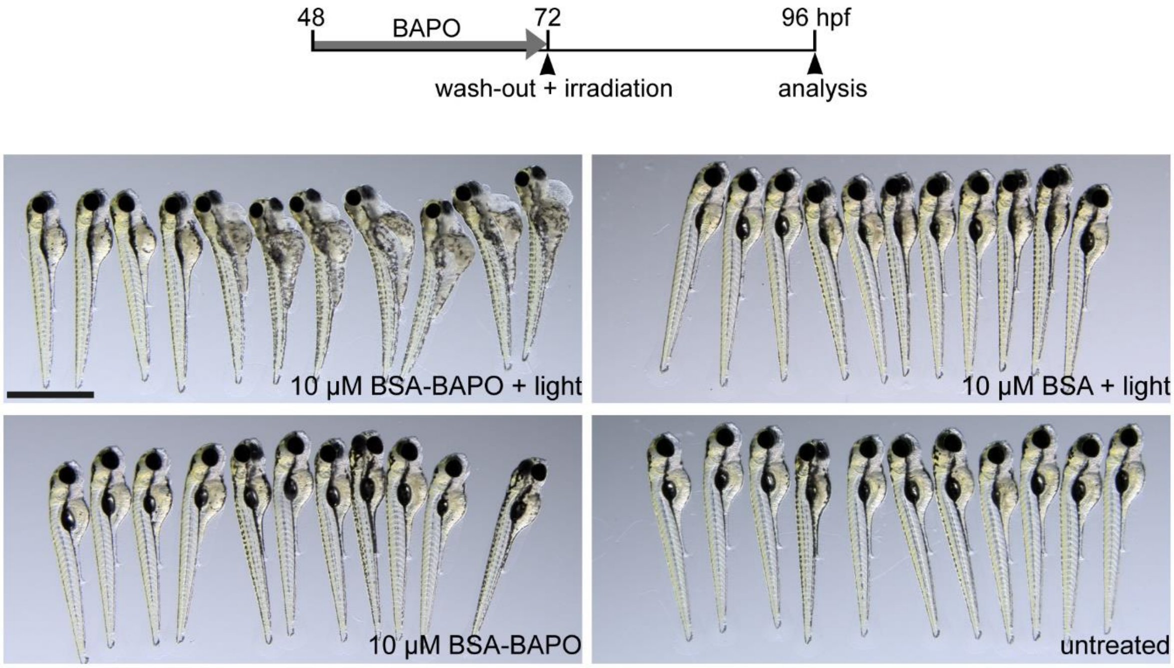

Figure S15: Zebrafish larvae 24 h after BIOS induction. All larvae investigated are shown. Exacerbated loss of tissue integrity and severe cardiac edema are visible for larvae subjected to BIOS. Control conditions are unaffected. Scale bar: 1 mm.

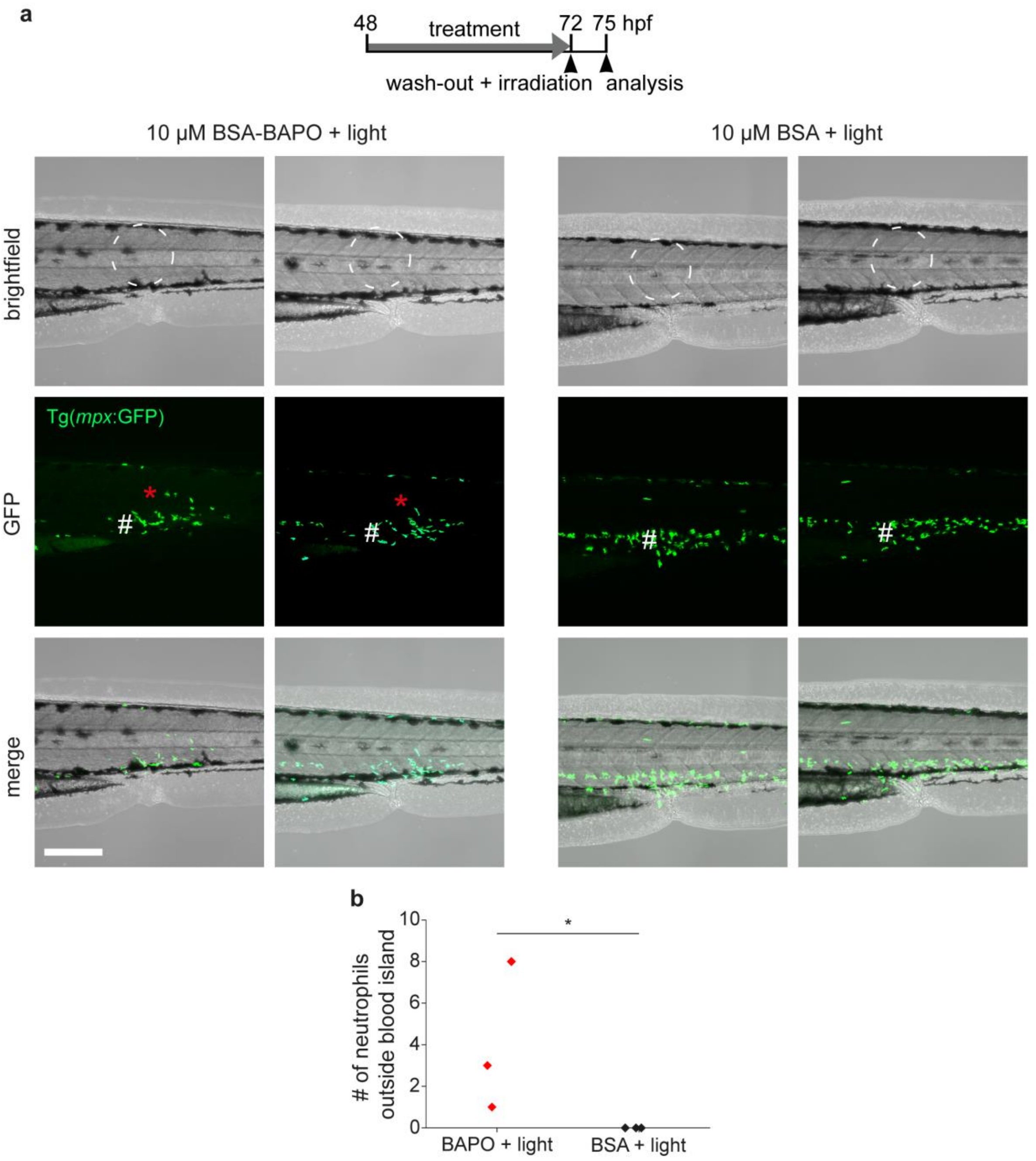

Figure S16: Local induction of BIOS in zebrafish larvae. (a) Neutrophil recruitment (red asterisks) is observed in the irradiation area (white circle). Neutrophils are shown in green fluorescence´. White hash signs denote neutrophils in the caudal hematopoetic tissue where they normally reside. (b) Quantification of neutrophils outside the caudal hematopoetic tissue. Mann-Whitney-Wilcoxon test was used to calculate the significance level. *: $p < 0.05$. Note the irradiation only control again contains unmodified BSA. Scale bar: 200 µm. Intensity: 9 mW/cm^2 for 30 min.

Supplementary Information on spectroscopic BAPO characterization

Glutamine BAPO

- ^{1}H NMR (500 MHz, $CDCl_3$): δ (ppm): 7.23 (d, JHH = 6.8 Hz, 1H, NH), 6.91, 6.88, 6.86 and 6.84 (s, 4H, H_{arom} Mes), 6.51 (d, JHH = 4.3 Hz, 1H, NH), 4.50 (q, JHH = 5.5 Hz, 1H, N-CH), 4.38 (q, JHH = 6.2 Hz, 1H, N-CH), 2.38, 2.32, 2.31 and 2.30 (s, 6H, p-CH_3), 2.29, 2.25, 2.20 and 2.19 (s, 12H, o-CH_3), 1.28 and 1.25 (d, 3H, CH_3). CH and CH_2 signals not assigned, several multiplets are in the region 1.9-2.9 ppm, some signals are under the CH_3 of the mesitoyl substituent.
- ^{31}P NMR (203 MHz, $CDCl_3$): δ (ppm): 25.5 (m, 2,4JPH = 10.7 Hz, O=PR_3), 24.1 (m, 2,4JPH = 9.2 Hz, O=PR_3).
- $^{31}P\{1H\}$ NMR (203 MHz, $CDCl_3$): δ (ppm): 25.5 (s, O=PR_3), 24.1 (s, O=PR_3).
- ESI-TOF (m/z) (+): 557.2411 (M+H)+.

Alanine-BAPO

- ^{1}H NMR (300 MHz, $CDCl_3$):δ (ppm): 6.90, 6.76, 6.74 (s, 4H, H_{arom} Mes), 6.68 (s, 1H, NH), 3.21 (br, 1H, N-CH), 2,71 (br, 2H, CH_2), 2.31 (s, 3H, CH_3), 2.21, 2.14 (s, 6H, p-CH_3 and o-CH_3), 1.07 (br, 3H, CH_3). CH signal not assigned.
- $^{31}P\{1H\}$ NMR (121 MHz, $CDCl_3$): δ (ppm): 25.4 (s, O=PR_3).
- ^{31}P NMR (121 MHz, $CDCl_3$): δ (ppm): 25.4 (br, O=PR_3).

GABA-BAPO

- ^{1}H NMR (500 MHz, $CDCl_3$): δ (ppm): 6.88 and 6.86 (s, 4H, H_{arom} Mes), 6.66 (s, 1H, NH), 3.23-3.16 (m, 1H, CH_2), 3.13-3.07 (m, 1H, CH_2), 2.82-2.77 (m, 1H, CH_2), 2.76-2.69 (m, 1H, CH), 2.35-2.32 (m, 1H, CH_2), 2.31 and 2.29 (s, 6H, p-CH_3), 2.26 and 2.22 (s, 12H, o-CH_3), 2.23-2.19 (m, 2H, CH_2), 1.77-1.63 (m, 2H, CH_2), 1.24 (d, $^{3}J_{HH}$ = 6.5 Hz, 3H, CH_3).
- ^{31}P NMR (203 MHz, $CDCl_3$): δ (ppm): 24.6 (m, $^{2,4}J_{PH}$ = 10.7 Hz, O=PR_3).
- $^{31}P\{1H\}$ NMR (203 MHz, $CDCl_3$): δ (ppm): 24.6 (s, O=PR_3).
- $^{13}C\{1H\}$ NMR (126 MHz, $CDCl_3$): δ ppm): 214.5 (d, $^{1}J_{CP}$ = 53.2 Hz, P-C=O), 214.2 (d, $^{1}J_{CP}$ = 52.2 Hz, P-C=O), 176.0 (s, COOH), 174.8 (d, $^{3}J_{CP}$ = 5.0 Hz, N-C=O), 141.8 and 141.5 (d, C^4 Mes), 136.3 and 135.6 (s, $C^{2,6}$ Mes), 135.7 and 135.0 (d, $^{2}J_{CP}$ = 42.2 and $^{2}J_{CP}$ = 41.2 Hz, C^1 Mes), 129.3 and 129.3 (s, $C^{3,5}$ Mes), 38.8 (s, CH_2), 34.5 (d, $^{2}J_{CP}$ = 4.3 Hz, CH), 31.2 (s, CH_2), 29.6 (d, $^{1}J_{CP}$ = 52.2 Hz, CH_2-P), 24.6 (s, CH_2), 21.3 (s, p-CH_3), 20.2 (d, $^{3}J_{CP}$ = 4.0 Hz, CH-CH_3), 19.8 (s, o-CH_3).
- ESI-TOF (m/z) (+): 514.2353 $(M+H)^+$.

BSA-BAPO

- ^{1}H-NMR (300.1 MHz, DMSO-d6, 298 K): δ (ppm): 6.97 (s, 4H, H_{arom} Mes), 2.28 (s, 6H, p-CH_3), 2.12 (s, 12H, o-CH_3).
- $^{31}P\{^{1}H\}$-NMR (121.5 MHz, DMSO-d6, 298 K): δ (ppm): 26.43 (br, O=PR_3).
- ^{31}P-NMR (121.5 MHz, DMSO-d6, 298 K): δ (ppm): 26.44 (br, O=PR_3).

Synthesis of BSA-BAPO

In a 50 ml round bottomed flask the BSA (2.529 g, 0.038 mmol, 1 eq.) is dissolved in 30 ml of 0.25 M carbonate buffer at room temperature. The NHS-BAPO (0.300 g, 0.57 mmol, 15 eq.) is dissolved in 2 ml of DMSO in a vial protected from light. The solution of NHS-BAPO is added dropwise to the water solution. The mixture is stirred vigorously for 1 hour at room temperature and protected from light. The mixture is diluted adding 30 ml of deionized water, then it is filtered and dialyzed. The product BSA-BAPO is recovered after lyophilization of the dialyzed solution, and the solid product is grinded to obtain a fine pale yellow powder (Yield: 2.3 g).
